# Environmental drivers of body size evolution in crocodile-line archosaurs

**DOI:** 10.1038/s42003-020-01561-5

**Published:** 2021-01-07

**Authors:** Maximilian T. Stockdale, Michael J. Benton

**Affiliations:** 1 School of Geographical Sciences, University Road, Bristol, BS8 1RL United Kingdom; 2 School of Earth Sciences, Life Sciences Building, 24 Tyndall Avenue, Bristol, BS8 1TQ United Kingdom

**Keywords:** Biodiversity, Palaeontology

## Abstract

Ever since Darwin, biologists have debated the relative roles of external and internal drivers of large-scale evolution. The distributions and ecology of living crocodilians are controlled by environmental factors such as temperature. Crocodilians have a rich history, including amphibious, marine and terrestrial forms spanning the past 247 Myr. It is uncertain whether their evolution has been driven by extrinsic factors, such as climate change and mass extinctions, or intrinsic factors like sexual selection and competition. Using a new phylogeny of crocodilians and their relatives, we model evolutionary rates using phylogenetic comparative methods. We find that body size evolution follows a punctuated, variable rate model of evolution, consistent with environmental drivers of evolution, with periods of stability interrupted by periods of change. Regression analyses show warmer environmental temperatures are associated with high evolutionary rates and large body sizes. We confirm that environmental factors played a significant role in the evolution of crocodiles.

## Abstract (web summary)

Stockdale and Benton use a new phylogeny of living and extinct crocodilians and their closest relatives to model evolutionary rates and identify the relative influences of extrinsic and intrinsic drivers of evolution in this order. Their results show that environmental factors played a significant role in the evolution of crocodiles.

## Introduction

Crocodiles might be interpreted as something of an anachronism. The modern crocodilian body plan has existed since the Early Jurassic, 200 Ma^1^, and yet their extant diversity is only 24 species^2^. Other clades of similar antiquity, such as dinosaurs (including birds)^3^ and lepidosaurs^4^, have each achieved a diversity of over 10,000 species in the same time interval^5,6^. The living Crocodylia are limited to amphibious ambush predators, with an armoured, barrel-like body, sprawling or semi-sprawling posture and a powerful tail^2^. However, they have a rich fossil record that documents hundreds of species with diverse modes of life, and occupying a wide range of habitats and geographical distribution^7^. Here, we explore crocodylian macroevolution in the context of the wider clade to which they belong^8^, the Pseudosuchia, or Crurotarsi. This is the ‘crocodylian line’ of archosaur evolution, which split from the ‘bird line’, including dinosaurs, birds and pterosaurs, in the Early Triassic.

During the Mesozoic, the Pseudosuchia comprised a diverse array of species, including derived marine and terrestrial forms, large armoured herbivores and cursorial hypercarnivores^2^. By contrast, extant crocodilians all share a similar body plan and an amphibious mode of life. This loss of diversity and morphological and functional disparity has suggested to some that the living crocodilians are ‘living fossils’^9^, a relict clade that has faded away through geological time through declining evolutionary rates or a failure to adapt. The term ‘living fossil’ has multiple definitions. One is that their lineage includes many Lazarus taxa representing gaps in the fossil record^10^. An alternative definition is bradytely^11^, namely a decline in rates of evolution; this may be an explanation for why crocodylians have faded from great biodiversity to relatively low species richness today. Herrera-Flores and colleagues^12^ define a living fossil as a taxon that shows below-average evolutionary rates and morphological conservatism, variables, together with bradytely, that can be tested using phylogenetic comparative methods (PCM).

Simpson^10^ introduced the idea of a deep-time evolutionary rate, which can be considered as phenotypic change per unit time. PCM enable these rates to be modelled through the reconstruction of ancestral states using a phylogenetic tree. PCM require character (trait) data in order to sample evolutionary change. Body size is commonly used as a trait in these analyses^13–16^ because it determines aspects of physiology, population size, resource consumption, geographic range, growth rates, life history, and reproductive success^17–20^, and can be measured in fossils. There is a tendency in many clades for body size to become larger through time, sometimes termed Cope’s Rule^21,22^. Larger animals may be more vulnerable to extinction than smaller ones, requiring more food and taking longer to reach sexual maturity^21^. However, the ubiquity of Cope’s Rule has been questioned^21^. Such an increase in body size may be a statistical artefact arising from increasing diversity, or it may represent a passive change, rather than an active trend.

The extent to which evolution is driven by intrinsic ecological interactions or extrinsic environmental changes is a matter of debate. The Red Queen hypothesis^23^ suggests that evolution is driven by intrinsic factors, such as competition, sexual selection, parasitism and the arms race between predators and prey. The Court Jester hypothesis proposes that evolution is driven by environmental changes, such as long-term fluctuations in temperature, atmospheric composition or sea level, or episodic shock changes caused by massive volcanism, bolide impacts and plate tectonics^24^.

Environmental change is of particular relevance to the Pseudosuchia. Since the Late Triassic, the Pseudosuchia have survived multiple dramatic environmental shifts coinciding with the end-Triassic and end-Cretaceous mass extinctions^2,8^, the Paleocene-Eocene Thermal Maximum, the end-Eocene extinction event, and cooling throughout the later Cenozoic. Recent work suggests that crocodilian diversity depended on climate. The geographic range of crocodilians is limited by environmental temperature because of their ectothermic physiology^25^. One study identified a linear relationship between diversity of crocodylomorphs and sea surface temperature, but this relationship was not observed in the Thalattosuchia^26^. Another study^27^ identified a linear relationship between loss of terrestrial crocodylomorph diversity and aridification of the climate. However, in this study amphibious taxa were placed among land-dwelling forms rather than comparable aquatic forms, and a different classification might change the results.

Phylogenetic approaches to diversification identified dynamic evolution in pseudosuchian subtaxa^28^. The clade Pseudosuchia includes major Triassic groups such as Phytosauria, Aetosauria, Rauisuchidae and Poposauroidea, as well as the Crocodylomorpha. Diversification rate shifts have been observed near the base of the Crocodyomorpha, Crocodyliformes and Neosuchia during the Late Triassic and Early Jurassic^28^. Similar shifts are seen in the Metriorhynchidae and Goniopholididae in the Early Cretaceous and in the Alligatoridae in the Paleocene. A recent analysis^29^ used PCM to identify different regimes of body size evolution localised to subgroups within Crocodylomorpha. Differences from our results are discussed below in terms of methods and base phylogenies, especially that the phylogenetic position of *Tomistoma* based on molecular phylogenetics^30^ was not incorporated into these previous studies.

In this study we use a new species-level phylogeny and PCM to investigate drivers of body size macroevolution in pseudosuchian archosaurs. We reconstruct evolutionary rates and derive a phylogenetic model of body size evolution in pseudosuchians. This phylogenetic model gives insights into the tempo and mode of crocodile-line archosaur evolution through time, which is considered in the context of the Red Queen and Court Jester hypotheses. Phylogenetic models are corroborated with time-series representations and linear models of body size against an environmental variable. Together, these approaches identify the respective roles that intrinsic biological and extrinsic environmental factors had in driving the evolution of the Pseudosuchia.

## Results and discussion

### Evolutionary rates through phylogeny and through time

**Fig. 1:**
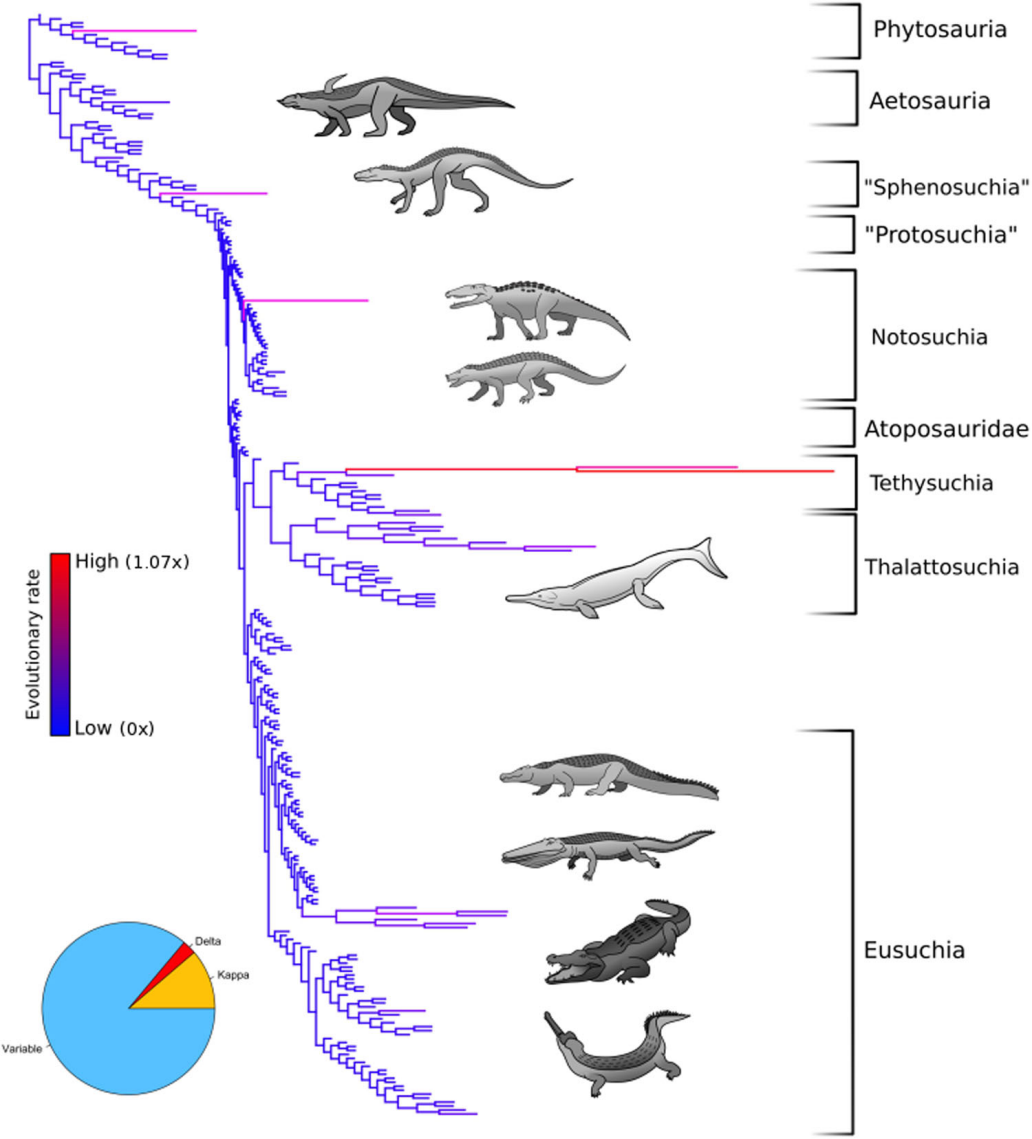
Rates of body size evolution in the Pseudosuchia. Phylogeny of the Pseudosuchia with branch lengths set to indicate evolutionary rate. Long branches, shown in red, indicate high evolutionary rate. Short branches in blue indicate lower evolutionary rates. The rate scalars were output by a variable rate phylogenetic model implemented using BayesTraits.

The phylogenetic tree (Fig. 1) shows reasonably uniform rates throughout, except for seven species whose evolutionary rates are high, namely the phytosaur *Angistorhinus*, the basal crocodylomorph *Carnufex*, the notosuchian *Razanandrongobe*, the elosuchids *Sarcosuchus* and *Terminonaris*, the teleosaurid *Machimosaurus* and the eusuchian *Purussaurus*. All of these are large animals, and they mostly occur in the Mesozoic, except for *Purussaurus*. The Bayesian analysis of body size shows that no larger clades showed bursts of either small or large body size. Evolutionary rate shifts do not appear to be associated with specific phylogenetic groups. This contrasts with the findings of previous studies, which showed that certain monophyletic clades follow a distinct model of body size evolution^29^. This difference may be attributable to the choice of body size proxy. The previous analysis made use of skull length as a body size proxy^29^, but this is dependent on overall skull shape; there are short-snouted and long-snouted crocodilians, and so a single skull length might correspond to animals whose body lengths differ by a factor of two and whose body masses differ by as much as an order of magnitude. Therefore the results of this previous analysis^29^ might be driven by the phylogenetic signal of skull shape, rather than a change in the tempo and mode of body size evolution.

**Fig. 2:**
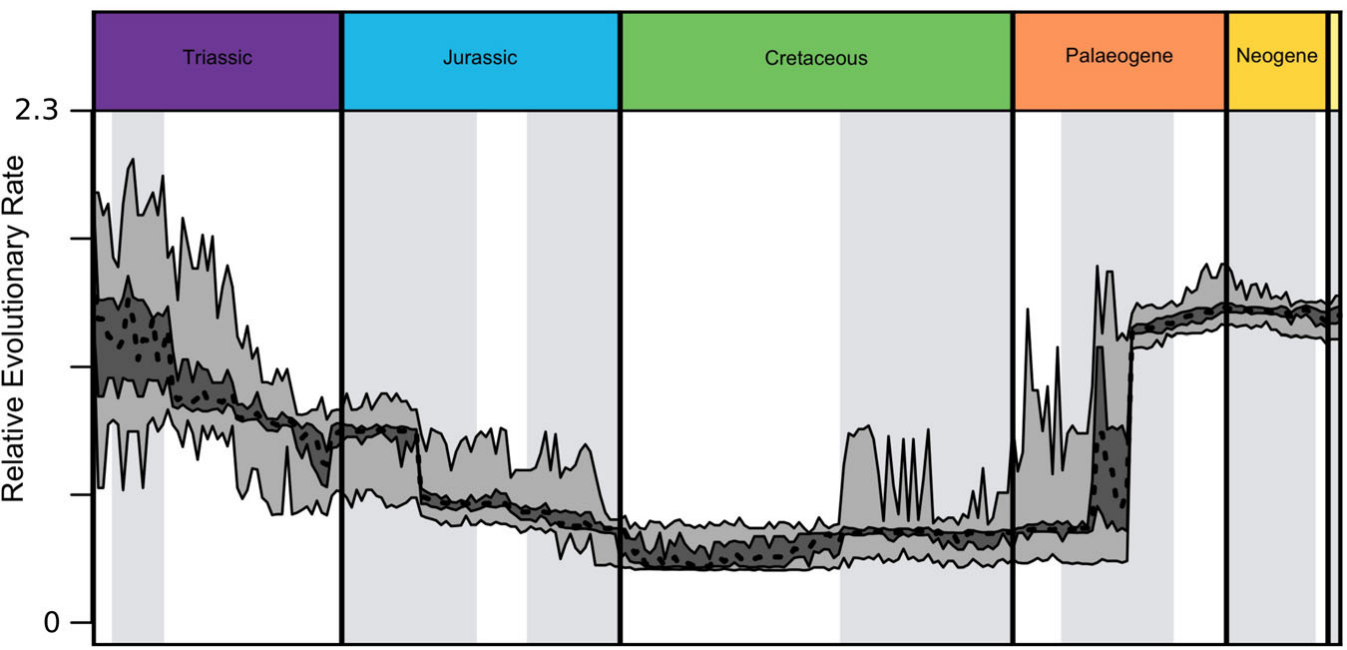
Time-series representation of evolutionary rates. Rate values were output by a variable rate phylogenetic model implemented using BayesTraits. The taxa included in each 1-million-year interval are denoted using a phylogenetic tree, dated using using first and last appearance dates. The taxa sampled in each 1-million-year interval were bootstrapped. Each bootstrap corresponded to a random 50% sample of the data in each bin, repeated 100 times. The interquartile range is shown in dark grey, the total range in light grey.

When plotted against geological time, the time series of mean evolutionary rates (Fig. 2) shows a stepped pattern, with long episodes of unchanging rates broken periodically by very rapid changes. Rates are high throughout the Triassic, but they decline through this period, levelling out through the Early Jurassic, and stepping down to lower rates in the Middle and Late Jurassic, and reaching their lowest value in the Early Cretaceous. These low values continue into the Paleogene, but with increased spikiness of the upper error limit through Late Cretaceous and Paleogene. Across the Cretaceous–Paleogene boundary, the instability of rates increases, but the mean rate remains constant. Rates step up sharply during the Eocene, and retain steady higher values, similar to those of the Middle Triassic, through the Oligocene and Neogene.

### Crocodiles are not ‘living fossils’

**Fig. 3:**
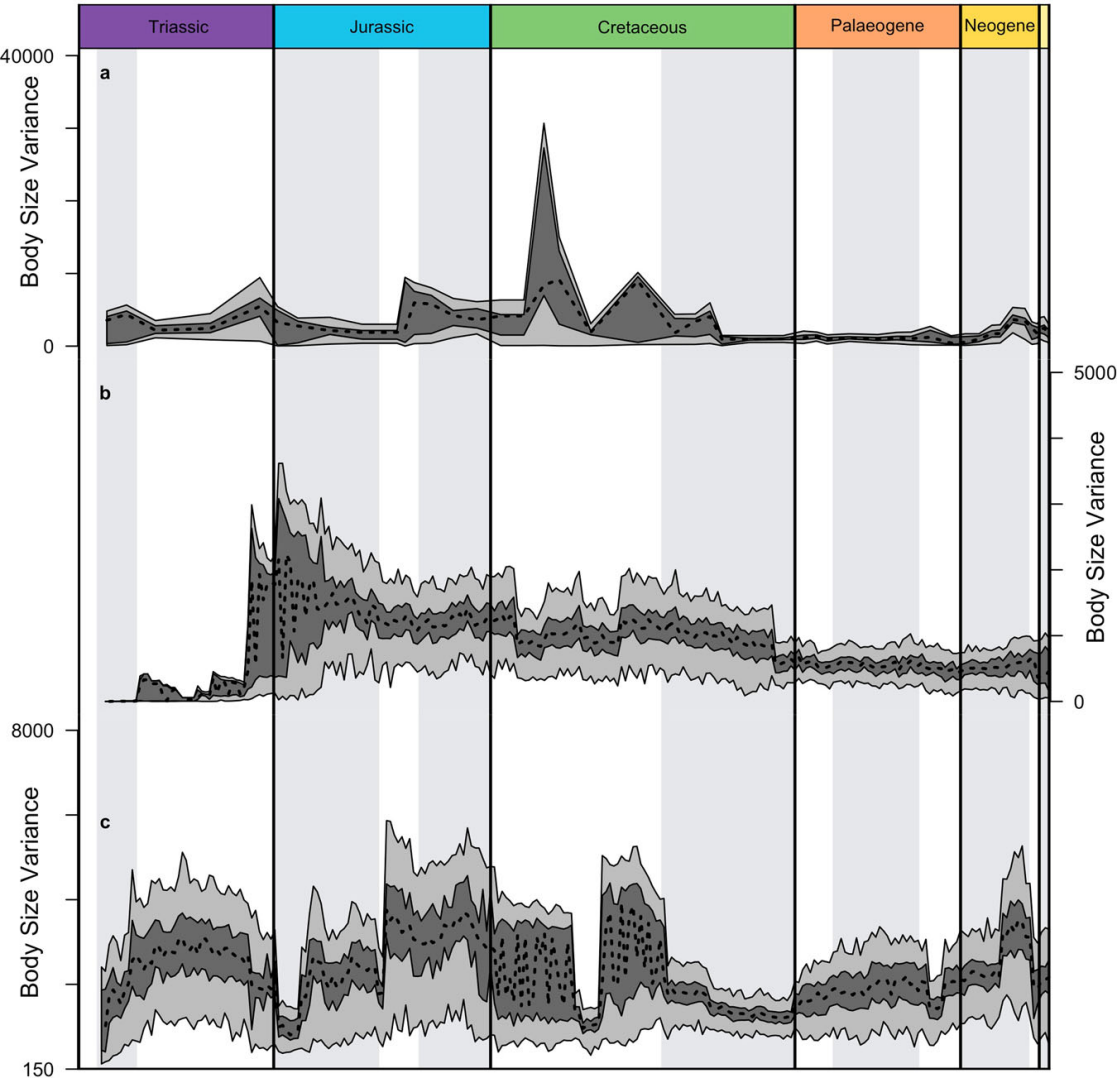
Time-series representations of relative body size variance. Three approaches are shown, as in Fig. 4: (**a**) actual taxa binned at the resolution of stratigraphic stages; (**b**) actual taxa plus ghost ranges inferred using branch lengths; (**c**) actual taxa plus ghost ranges plus phylogenetically reconstructed body size values of inferred common ancestors. The taxa sampled across all methods were subjected to bootstrapping; the interquartile range is shown in dark grey, the total range in light grey.

Contrary to previous publications^9^, we do not find any evidence that living crocodilians are living fossils. One definition of a living fossil is as a ‘Lazarus taxon’, meaning that extant examples exist despite a prolonged absence from the fossil record, for example the coelacanth *Latimeria*. The crocodilians cannot be described as Lazarus taxa, since fossil members are known throughout the Mesozoic and Cenozoic eras. Bradytely is an alternative definition^11^, in which living fossils are described as taxa that have shown a decline, and eventual stagnation, of their evolutionary rates. The study of evolutionary rates provides no justification to describe extant crocodilians as bradytelic, since evolutionary rates of extant species are shown to be neither low nor decreasing (Figs. 1, 2). A further definition of living fossils is that they show low evolutionary rates combined with morphological conservatism^12^. It is true that the body size disparity of extant crocodilians is relatively low (Fig. 3), irrespective of which method is used to reconstruct the time-series. However, as noted above, the evolutionary rate of the crocodilians is surprisingly high, particularly in the Neogene (Fig. 2).

### Relative body size through time

**Fig. 4:**
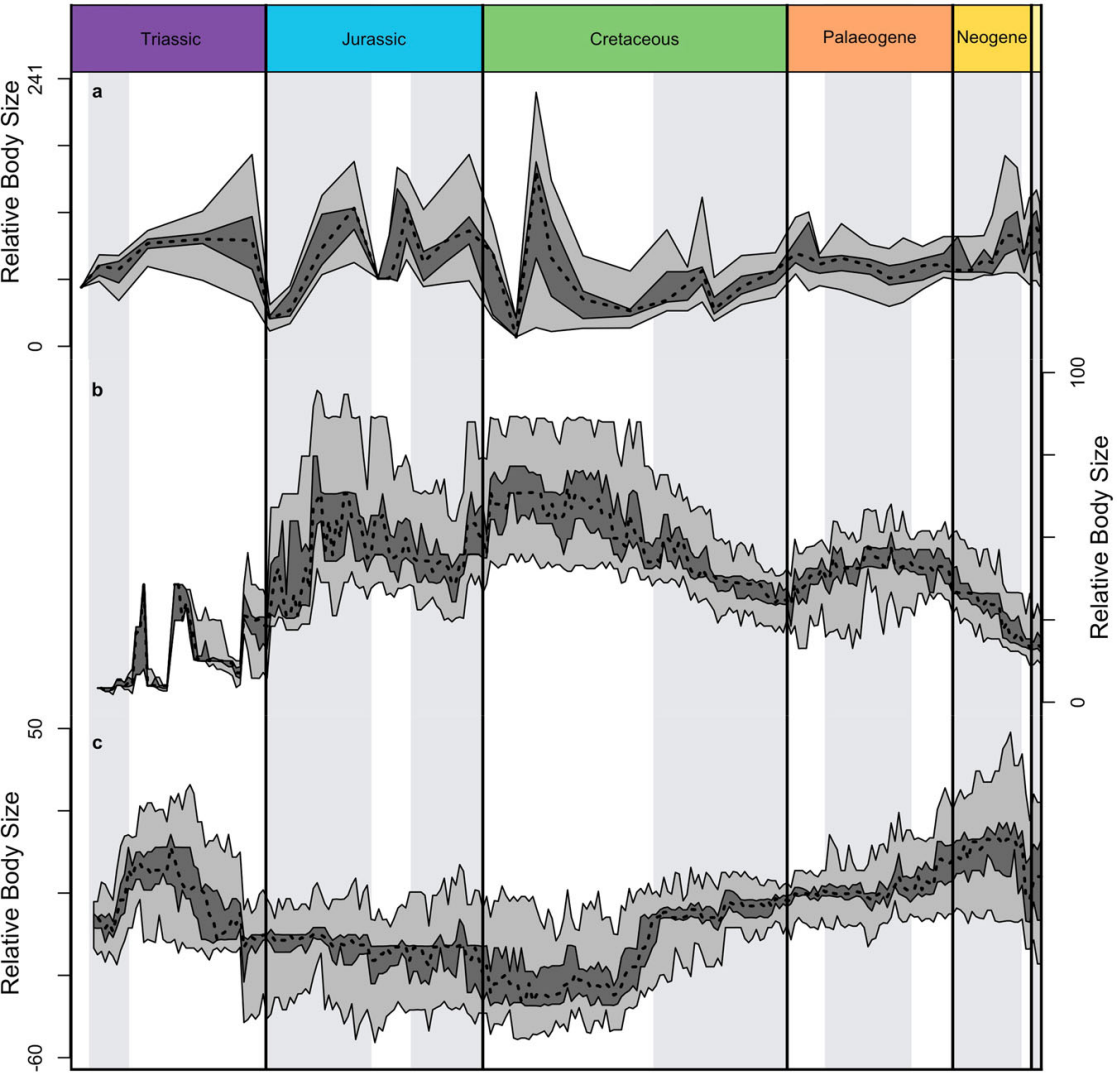
Time-series representations of mean relative body size. Three approaches are shown: (**a**) actual taxa binned at the resolution of stratigraphic stages; (**b**) actual taxa plus ghost ranges inferred using branch lengths; (**c**) actual taxa plus ghost ranges plus phylogenetically reconstructed body size values of inferred common ancestors. Random 50% bootstrap samples were drawn over 100 iterations. The interquartile range of these iterations is shown in dark grey, the total range in light grey.

The fossil record is assumed to be highly incomplete, and subject to significant preservation bias. In order to take these biases into account, our analysis employed three different approaches to reconstructing time series of body size and disparity. This included an empirical approach, which assumes the fossil record is representative of the true pattern, and two derived methods using the phylogenetic tree to infer missing taxa (see “Methods” section). These three methods give differing results (Figs. 3, 4). The empirical time series (Fig. 4a) shows constant change in relative body size, with rapid switches from positive to negative and back again, but a seemingly similar base level throughout. Empirical body size variance (Fig. 3a) is relatively steady, but with a sharp high value in the Early Cretaceous.

The time series with ghost ranges (Fig. 4b) has a generally smoother profile and follows sweeping curves, with rising values through Triassic and Jurassic, constant in the Early Cretaceous, and falling through the Late Cretaceous, rising slightly in the Paleogene, and falling again in the Neogene. The distribution of variance (Fig. 3b) shows low values in most of the Triassic, with the maximum value rising sharply in the Late Triassic, and then the range of variance narrowing from a widest range in the Early Jurassic through to the present day, while mean values fall gradually through the same time interval.

The time series with ghost ranges and ancestral taxa (Fig. 4c) shows the opposite pattern, with body size values falling from Triassic highs to steady low values through the Jurassic and Early Cretaceous, and then stepping up at the beginning of the Late Cretaceous, and then rising slowly through the Palaeogene and early Neogene, before falling in the Pliocene and Pleistocene.

The substantial difference between the three time-series can be attributed to the amount of missing taxa. The patterns of change in body size seen in the empirical time-series are evidently driven in large part by the quality of the fossil record. This may arise from the relative preservation potential of small and large specimens, periods of erosion and the occurrence of fossil lagerstätten. Inferring taxa in a given time-bin using phylogeny may adjust for taxa missing from the fossil record. The difference between the two phylogenetically adjusted time-series indicates that the absence of common ancestors from the fossil record is a further driver of the empirical pattern, in addition to known taxa that are missing from parts of their temporal range.

The distribution of variance (Fig. 3c) shows reasonably steady mean values throughout, but with some steps in the Middle Jurassic, Palaeogene and Neogene, and considerable instability through the Cretaceous. Maximum values fluctuate substantially, with highs through much of the Mesozoic, except for lows after the end-Triassic mass extinction and in the Early Cretaceous, and with lows generally through most of the Late Cretaceous, Palaeogene and Neogene.

A key question concerning plots of this kind is whether they document reality or heterogeneity of sampling. We can address this conundrum to some extent by comparing the three plots in Figs. 3, 4. For example, the time series without ghost ranges or common ancestors (Figs. 3a, 4a) are in a state of constant change, with the direction of that change shifting frequently. On the other hand, the time series including ghost ranges (Figs. 3b, 4b) and including ghost ranges and common ancestors (Figs. 3c, 4c) show some smoothing of the curves, which might reflect the plugging of some gaps in the fossil record by inferred data.

### Evolutionary model fitting

All the fitted phylogenetic models achieve a uniform likelihood distribution (Supplementary Data 1), indicating that they reached convergence. The variable rate model, the kappa model and the delta model all significantly outperformed the random walk according to their Bayes factors, but the directional trend, lambda and Ornstein–Uhlenbeck models did not (Table 1).

**Table 1:** Output parameters from each of the phylogenetic models implemented in BayesTraits.

Model	Log Bayes factor compared with random walk	σ^2^	α	Statistic
Random walk	N/A	43.095	−11.705	N/A
Lambda	−3.337	43.184	−11.561	λ = 0.978
Delta	5.183	41.803	−11.823	δ = 0.735
Kappa	22.200	33.156	−14.337	κ = 0.395
Variable rates	169.658	41.285	−14.148	No. of rates = 30.5
Ornstein–Uhlenbeck	−3.996	44.002	−11.869	OU = 0.000173
Directional trend	−1.009	43.252	−12.064	β = 0.0151

Phylogenetically adjusted mean body size α differs between each of the models fitted (Table 1). A weighted mean of the α-values returned by the kappa, delta and variable rates models finds a PC-1 value of −14.108. This corresponds to an estimated skull width of approximately 15 cm. The arithmetic mean of PC-1 gives a PC-1 value of 0.782, corresponding to a skull width of approximately 18 cm.

We note that skull width alone would not be suitable as a body size metric, because there is considerable diversity of relative head sizes among the Pseudosuchia. However, the skull widths predicted by the arithmetic mean of PC-1 scores and the phylogenetically adjusted α-values returned by the phylogenetic models do provide interesting insights. As noted, the arithmetic mean of PC-1 values corresponds to a skull width 3 cm wider than the phylogenetically adjusted estimate. This suggests that the distribution of trait data is skewed towards larger specimens. Bias towards greater size is not surprising in fossil data, since larger specimens tend to be more resistant to decay and erosion. Including phylogenetically reconstructed common ancestors may help to mitigate this issue in the context of time-series reconstructions of trait values.

We find that pseudosuchian body size is not constrained near an optimum value, because the Ornstein–Uhlenbeck (OU) model of body size evolution does not perform significantly better than a random walk (Table 1). Concerns have been raised about the susceptibly of the OU model to false positive results, being difficult to distinguish from the random walk model^31,32^. The recommendation^31,32^ is that OU models should only be fitted to samples of 200 or more observations, and only using Bayesian methods. Here, we satisfy both of these criteria, and the OU model is not selected as a likely explanation. We cannot say that stabilising selection has not taken place at any point during the course of pseudosuchian body size evolution, but it is not the best explanation for the whole trajectory. These findings conflict with a recent study^29^, in which an OU model was the best supported, but was particularly applicable to certain groups. Further, this previous analysis^29^ implemented the *SURFACE* model, which appears to suffer from high rates of false positives^33^.

In our study, the lambda model fails to perform significantly better than a random walk (Table 1). The lambda model attributes trait variation to phylogeny, and cursory observation shows that most clades comprise a mixture of large and small species. For example, the Crocodylidae includes the relatively small *Osteolaemus tetraspis* and the giant *Crocodylus thorbjarnarsoni*. Similarly, the phylogenetically distant Notosuchia includes small genera such as *Araripesuchus* and much larger animals like *Razanandrongobe*. Therefore, changes in body size take place independently of phylogeny.

Earlier work^34,35^ has shown that the Pseudosuchia diversified rapidly during the Triassic. We corroborate these findings, especially by identifying that the delta model fits significantly better than a random walk (Table 1). A delta value of less than 1, as found here (0.74), indicates that phenotypic change is concentrated at the start of a phylogenetic tree branch^36^. We find an increase in pseudosuchian mean body size during the Triassic, but differing according to the input data, either rising irregularly through the Triassic (Fig. 4b), or rising in the Early and early Middle Triassic before levelling (Fig. 4a) or falling (Fig. 4b) in the Late Triassic. Similar to body size, the time series including ghost ranges and common ancestors shows the greatest agreement with the delta model (Fig. 4c).

The evolutionary rate increase associated with the Middle Jurassic notosuchian *Razanandrongobe* is exceptional, especially when compared to the relatively low evolutionary rates observed among other notosuchians. *Razanandrongobe* has been described as an island-dweller, with a fossil range coinciding with the separation of Madagascar from mainland Africa^37^. Increased evolutionary rate among island species is well documented^38^. Perhaps *Razanandrongobe* is an example of island gigantism, and its size not representative of Jurassic notosuchians in general.

It is apparent from the variable rate model that the marine Thalattosuchia and Tethysuchia experienced an evolutionary regime that was distinct from that of most other Pseudosuchia, which occupied terrestrial or freshwater habitats. Evolutionary rates of internal branches within these clades are relatively high, more so than the majority of other neosuchian clades. This may support the idea of the radiation of Thalattosuchia into vacant marine niches^39^. Other aspects of thalattosuchian biology have also attracted controversy; Young and colleagues^40^ speculated that the metriorhynchid Thalattosuchia might have been viviparous, based on the width of the pelvic girdle. Adopting viviparity could have freed the metriorhynchids from the limitations of laying eggs on land, enabling them to develop a more hydrodynamic body plan. Martin and colleagues^26^ found the diversity of thalattosuchians to be incongruent with sea surface temperature, unlike other crocodylomorph groups. These authors proposed that the Thalattosuchia maintained a higher rate of metabolism than other clades. While viviparity and endothermy among thalattosuchians remains speculative, the interaction between physiology, reproductive strategies and evolutionary rates would be an interesting line of future research.

### Cope’s rule and the Pseudosuchia

These plots show little evidence for Cope’s rule. The time series of body size through time do not show an overall increase regardless of which reconstruction method is used (Fig. 4). The empirical time series (Fig. 4a) shows no net size change through the entire span of crocodilian evolution, that with ghost ranges (Fig. 4b) suggests a continual decrease in body size since the Early Cretaceous, and that with ghost ranges and common ancestors (Fig. 4c) shows a long decline in mean body size for 100 Myr, and then a step up to a slightly increasing trend through the Late Cretaceous and Cenozoic. This is clearly a limited episode of body size change, rather than an on-going trend. Among the fitted phylogenetic models, there is no support for the directional trend model compared to a random walk (Table 1). Therefore, a directional shift in pseudosuchian body size cannot be justified, either from small-bodied to large-bodied forms, as proposed by Cope’s rule, or vice versa. We identify equal numbers of shorter-term episodes of increasing and decreasing mean body size, and there is no evidence for Cope’s Rule through pseudosuchian history.

There are some conceptual issues around Cope’s rule, since the definition of any clade is arbitrary. Increases in body size may be attributable to other mechanisms, for example selection by external drivers. Alternatively, maximum body size may increase passively through an increase in diversity over time;^13^ in such cases, minimum and average body size need not increase if there is a corresponding rise in numbers of small-bodied taxa. Therefore, the periods of increasing average body size shown in the time-series suggest an episodic external driver of body size, rather than a passive increase. Maximum body size may have increased passively during periods when average body size was stable.

### Punctuated model of evolution

We have found multiple lines of evidence that pseudosuchians followed a punctuated mode of body size evolution rather than a gradual one. By far the best supported of the phylogenetic models fitted is the variable rate model. The evolutionary rate scalars returned by this model indicate that the Pseudosuchia have a stable background rate of body size evolution which is interrupted by short periods of higher evolutionary rates (Figs. 1, 2), and the highest rates are observed in individual species rather than larger branches. The kappa model does not perform, as well as the variable rate model, but it performs significantly better than a random walk (Table 1). This corroborates a punctuated rather than gradual mode of body size evolution. The kappa model fitted in this analysis returned a kappa statistic of 0.4. Kappa values of less than 0.5 indicate a punctuated model of evolution^36^, with changes in trait values being associated with cladogenesis. Higher kappa values indicate gradual evolution, or anagenesis. However, a kappa value of more than zero does not exclude a gradual component entirely. While this analysis suggests that the majority of variation has arisen through a punctuated model, it is possible that gradual change might have driven body size variation to a limited extent.

The time-series representations of body size vary in how closely they concur with a punctuated model (Fig. 4). The empirical time series seems to be in a state of permanent transition, with successive, rapid shifts between body sizes (Fig. 4a), while the time series curve with ghost ranges shows a smoother topology (Fig. 4c). Body size shifts in the empirical time series may be attributable to variation in the fossil record. The time series including ghost ranges and inferred common ancestors concurs more closely with the phylogenetic models, with long periods of stability throughout the Jurassic, in the Early Cretaceous, and through the Late Cretaceous to the Eocene–Oligocene boundary. These periods of stability are separated by discrete events where body size increases or decreases sharply (Fig. 4c).

The time series of body size disparity show similar patterns to that of mean body size (Fig. 3). Without ghost ranges or inferred common ancestors, body size disparity changes constantly between high and low values (Fig. 3a), but is fairly constant from the Late Cretaceous to mid-Miocene. The disparity time series with ghost ranges (Fig. 3b) is relatively smooth but punctuated by some small shifts during the Cretaceous. Disparity with both ghost ranges and common ancestors (Fig. 3c) also has a stepwise topology, but periods of stability are shorter and shifts in disparity are larger.

### Environmental drivers of crocodilian evolution

A punctuated model of body size evolution suggests that extrinsic environmental factors were the principal drivers, as predicted by the Court Jester hypothesis. Such changes might include mass extinctions, climate shifts or sea level changes. Some of the jumps in mean body size correspond to identified events in Earth history. For example, the downshift in relative body size in the Late Triassic at about 232 Ma (Fig. 4b, c) could correspond to the Carnian Pluvial Episode, but the empirical curve (Fig. 4a) shows a later drop in mean size. Two of the plots (Fig. 4a, b) show sharp shifts in mean body size across the Jurassic-Cretaceous boundary, a putative extinction event^39^, but this is not shown in the plot with ghost ranges and common ancestors (Fig. 4c), so some of the evidence for the event might be artificial. Surprisingly the Cretaceous-Palaeogene boundary does not appear to be associated with any changes in mean body size. In all cases, even with ghost ranges reconstructed, we may miss earlier unpreserved portions of the records of lineages^40^.

The time series of disparity including ghost ranges and estimated common ancestors shows disparity changes at many of the same points (Fig. 3c). This time series identifies additional shifts not visible from mean body size (Fig. 4c). A sharp drop in disparity is observed in the Carnian, at the Jurassic-Cretaceous boundary, at the Early-Late Cretaceous boundary, and after the end-Eocene extinction event. A second substantial drop in disparity is seen at the Triassic-Jurassic boundary, matching one of the most severe of all mass extinction events. There is also a modest increase in body size disparity following the Cretaceous–Palaeogene extinction event. These patterns in disparity further indicate that mass extinctions were a major influence on body size evolution. Changes in disparity also coincide with other significant events. There is an increase in disparity at the start of Miocene climate optimum, and a steep drop in disparity at the end of the Miocene climate optimum.

The Neogene fossil record, from 23 Ma to the present, shows evidence for interactions between temperature and body size. The size transitions, whether up (Fig. 4a, c) or down (Fig. 4b), appear to be gradual and they are associated with the Miocene Climate optimum.

Linear regression analyses of both body size and evolutionary rate with paleotemperature proxy data find a significant negative correlation (Table 2). The oxygen isotope paleotemperature scheme scales inversely with temperature^29^, therefore larger body sizes and higher evolutionary rates are associated with warmer temperatures. A direct relationship between temperature and body size would suggest that during warm periods crocodiles would be able to invest more resources into growth. Perhaps high temperatures enabled individuals to remain active for longer, and therefore able to spend a greater fraction of their time feeding. Warmer temperatures would be expected to increase the ecospace available to crocodiles and their relatives. Therefore, increased evolutionary rates during warm periods may reflect the radiation of taxa into new ecological niches.

**Table 2:** Outputs of linear regression models comparing isotope temperature proxies with three body size variables.

Dependent variable		Slope	R^2^	*p*-Value
Cenozoic	Mean body size	−1.76	0.553	2.076 × 10^−11^
	Body size variance	−70.76	0.258	3.293 × 10^−5^
	Mean evolutionary rate	−0.105	0.686	1.19 × 10^−15^
Mesozoic	Mean body size	−0.0416	0.206	5.77 × 10^−9^
	Body size variance	0.000238	0.0302	0.0205
	Mean evolutionary rate	−2.018	0.156	5.44 × 10^−7^

Linear regression analyses of body size, disparity, evolutionary rate and temperature in the Cenozoic recover a significant negative correlation (Table 2). The regression is weakest between temperature and variance, with an R-squared of just 0.25. The regression with body size is somewhat stronger, with an R-squared of 0.55. The best performing regression model is temperature with evolutionary rate, which achieves an r-squared of 0.69. Similar analyses of Mesozoic temperature also recover significant negative correlations for body size and evolutionary rate. However, the strength of the correlation is markedly lower, with R-squared values of 0.21 and 0.16, respectively (Table 2). Body size disparity and Mesozoic temperature show a positive correlation, but the strength of the correlation is negligible, and the p-value is only barely significant at 0.02. A low or modest R-squared value indicates that while there is a clear relationship between temperature and evolutionary rate, temperature alone is not a comprehensive predictor of evolutionary rate, body size or disparity. Therefore there is plenty of scope for other environmental variables, as well as intrinsic biological factors, to influence evolutionary rates. However, there is an apparent association between temperature and the majority of variation in evolutionary rates. The strength of correlation may also be influenced by biogeography. The time-series used in this analysis represents global averages, and does not account for regional variation in body size or temperature. Perhaps body size regimes varied between pseudosuchians in equatorial regions and cooler regions at higher latitudes.

The correlation of body size and evolutionary rate with temperature is considerably weaker in the Mesozoic than in the Cenozoic, perhaps because global temperatures were warmer and more stable in the Mesozoic^41^. Nevertheless, the correlation for both time intervals is negative, supporting a common mechanism throughout pseudosuchian history. This is in line with other published analyses that have indicated a relationship between the evolution of Mesozoic crocodile-line archosaurs and climate^25–27^.

The analyses presented in this study suggest extrinsic environmental factors played a significant role in driving the body size evolution of pseudosuchians. The phylogenetic models indicate that evolutionary rates have an uneven tempo, characterised by overall relative stasis punctuated by short periods of change. This is the model predicted by the Court Jester hypothesis, where evolution is driven by environmental changes^24^. Linear models of both body size and evolutionary rate corroborate this conclusion, showing a significant relationship with global temperature. Global temperature may not be the only driving factor, and there is plenty of scope for other factors to also play a role, including other environmental variables. Body size and disparity in crocodile-line archosaurs show changes associated with major events in Earth’s history, notably mass extinctions. Therefore, other major environmental shifts are likely to have played a significant role in pseudosuchian evolution.

## Methods

### Body size metrics

The masses of fragmentary fossilised remains are clearly not representative of the body mass of the animal in life. Body mass of fossil taxa is often represented in analyses by a linear metric that is considered to correlate with overall body size^13,14^. We could not determine full body length or snout-vent length in many fossil pseudosuchians because most lack complete skeletons. Skull length has been shown to be an indicator of body size in dinosaurs^41^. Skull length has been used in a recent study of crocodilian macroevolution^29^. These authors^29^ provided a linear regression of total skull length with total body length, but of the six taxa in their plot, the large-bodied examples are long-snouted, and the small-bodied examples are short-snouted. This is not a representative sample, since long-snouted and short-snouted forms are known across a range of body sizes; for example, a highly elongated snout is observed in both the modest-sized *Isisfordia duncani*^42^ and the giant *Sarcosuchus imperator*^43^. Likewise a short-snouted morphology occurs in the small-bodied *Shamosuchus djadochtaensis*^44^ and the exceptionally large *Razanandrongobe sakalavae*^37^. Therefore, this regression does not address the possibility that skull length may over-estimate body size in long-snouted taxa, and under-estimate it in short-snouted taxa. It is difficult to determine whether the patterns observed^29^ truly reflect the evolution of body size, or whether this pattern has been driven by the evolution of skull aspect ratios. An alternative measure, skull width, avoids contrasts of long-snouted and short-snouted forms, but would underestimate body size in small-headed forms such as aetosaurs.

The diameters of long bones have been used in body size studies in dinosaurs^14,15^. However, limb elements of pseudosuchians are not so abundant in the fossil record as skulls (Supplementary Data 1). Further, although the dimensions of a load-bearing skeletal element such as the femur or humerus are proportional to that load, non-avian dinosaurs were entirely terrestrial, with an exclusively erect gait. This is not true of the Pseudosuchia, in which some forms showed a sprawling or semi-erect gait^2^, and several clades of crocodylomorphs were partly or entirely aquatic^2^. These issues mean that femur length or diameter, for example, would not provide a reliable estimate of body mass or length across all taxa.

**Fig. 5:**
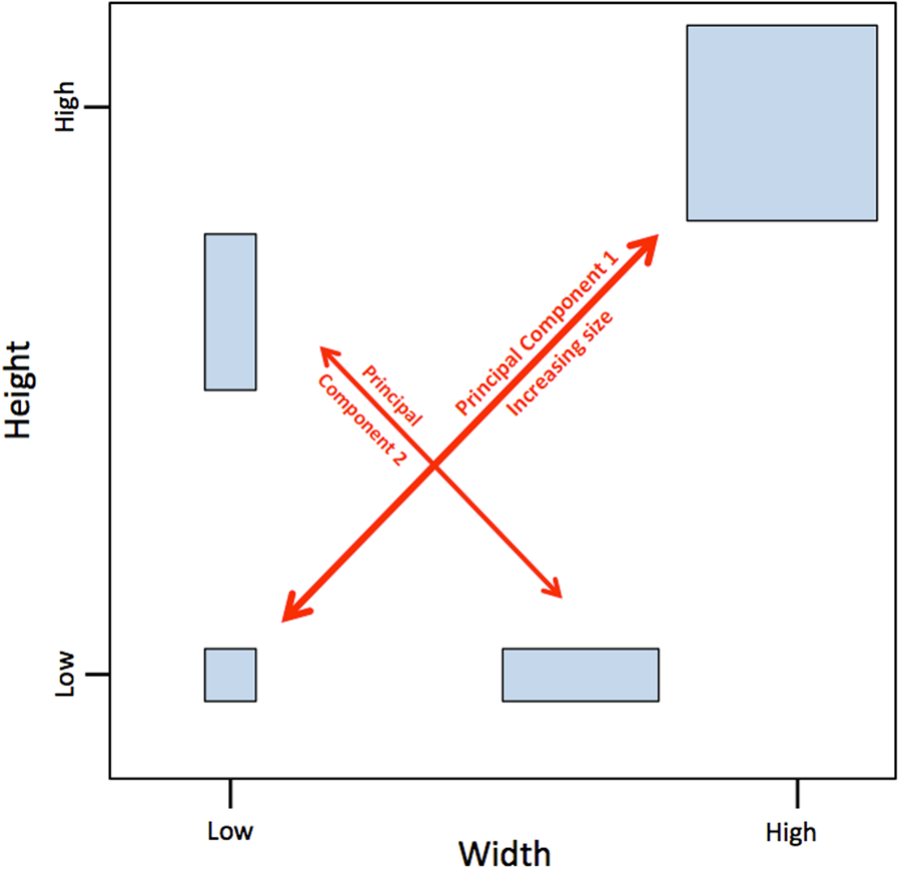
A simplified demonstration of how size can drive covariance between two characters. In this two-dimensional example, relative size is indicated by the position of points along the trend line, which corresponds to the first axis returned by principal components analysis. Variations in shape are indicated by position on the orthogonal second component. A positive correlation between traits may be driven by factors other than size, therefore this study uses 21 characters, rather than just two.

The body size proxy used in this analysis is the score of each taxon on the first component axis of a principal components analysis of 21 traits (Supplementary Data 1). This is based on the methodology of Smith^45^, which posits that variation in body size drives covariance across multiple traits. The analysis of Smith demonstrates that difference in size, or isometry, is indicated by the relative position of taxa on a trend line drawn through a bivariate space of two characters. Variation in body shape, or allometry, is indicated by the residual error of points above or below that trend line, and therefore this method is applicable to taxa of varying body proportions. A simplified demonstration of this rationale is shown on Fig. 5. Jolicoeur^46^ expands upon this concept into a multivariate model using principal components analysis. Principal components analysis re-orientates a multivariate dataset into a corresponding array of orthogonal axes, with each axis accounting for a decreasing fraction of variance. These axes are derived through correlation tests of each variable with each other variable. Strong correlations contribute more substantially to a principal component axis than a weak correlation. The positions of the first two principal components in a bivariate context are shown on Fig. 5. Principal components analysis commonly uses log-transformed data^47^ to standardise measurements and limit the effects of body size on interpretations of allometry. However, it has been observed that log transformations are not universally appropriate^48^, and in this study it is isometry, not allometry, that is of interest. Therefore standardising the data through log-transformation would be detrimental in this case. The approach of using principal components analysis to estimate relative body size has been applied in previous examples including birds^49^, bats^50^, rodents^51^, and insects^52^.

The dataset was assembled from the literature. Peer-reviewed articles featuring images of fossil specimens were sampled and measured using ImageJ^53^. Up to 21 characters were measured from each specimen, depending on their completeness (Supplementary Data 1). These included cranial, mandibular, humeral and femoral characters, selected to encompass the greatest diversity of body shape possible, without introducing an excessive amount of missing data. All the measurements were collected in centimetres. A non-parametric Spearman-rank correlation test was performed between all variables, and confirmed many positive correlations indicating positive covariance within the data (Supplementary Data 1). This covariance is likely to be driven by body size, because the variation within the data is extremely large. For example, skull width in the smallest and largest taxa differs by an order of magnitude. Therefore these data are suitable for body size estimation using principal components analysis.

**Fig. 6:**
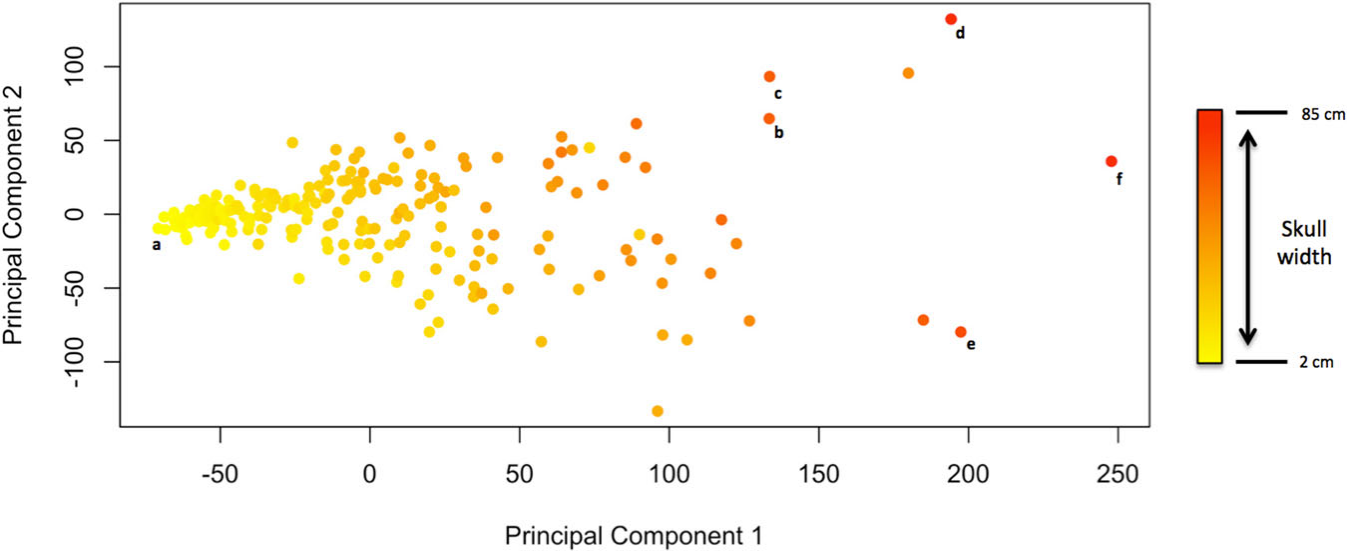
Ordination morphospace of the first two axes returned by the principal components analysis. Points are coloured according to relative skull width. This demonstrates the relationship between PC1 and body size, with larger taxa having higher PC1 scores. Note the colour gradient is not fully continuous along the *x*-axis, indicating taxa with disproportionately wide or narrow skulls. Notable taxa are indicated: (**a**) is the smallest taxon in the dataset, *Knoetsuchkesuchus guimarotae;* (**b**) *Razandrongobe sakalavae*, the largest notosuchian; (**c**) *Crocodylus thorbjarrsoni*, the largest crown crocodylid; (**d**) *Purussaurus brasiliensis*, the largest crown alligatorid; (**e**) *Machimosaurus*, the largest thalattosuchian and (**f**) *Sarcosuchus imperator*, the largest pseudosuchian.

The raw data were, necessarily, highly incomplete. The PCA was implemented with iterative imputation using PAST version 3.1 for Mac OS^54^. An advantage of this approach is that it is not limited by variable preservation of any single character. A dataset limited to only complete specimens would be vanishingly small, and unrepresentative of the clade as a whole. Likewise, using a single character as a body size proxy would limit the dataset to those specimens with that character preserved. This may introduce preservation bias, since the probability of preservation may vary between skeletal elements. Using iterative PCA enables relative body size to be estimated from multiple characters. If a given character is missing, its value is inferred from other characters that are preserved. Therefore, the analysis does not require any one character to be complete in every specimen, enabling relative body size to be estimated for a much larger number of taxa than would otherwise be possible. The final dataset included scores for a total of 280 taxa. The distribution of these 280 taxa along principal component 1 is shown on Fig. 6, including the position of some notable examples.

### Phylogenetic modelling

PCM studies of evolution require a phylogenetic tree. There is no published phylogenetic hypothesis that encompasses all Pseudosuchia, as well as molecular data from living taxa. Therefore, we estimated a new phylogenetic hypothesis for this study.

Previous studies of trait evolution in fossil taxa have been dominated by informal supertrees^29,55–61^. While informal supertrees have practical advantages, they have no underlying systematic basis and are therefore subjective. A matrix-based approach was also ruled out, because collecting character data for such a large matrix from the literature and vetting characters for redundancy would have been impractical. In addition, such a large matrix would have introduced a significant fraction of missing data, which could undermine the quality of a finished tree. The phylogenetic hypothesis used in this study is based on a formal supertree analysis (Supplementary Information). Formal supertrees use a systematic approach to assimilating multiple smaller topologies into a single tree. Such methods have been used previously in macroevolutionary analyses of fossil taxa^28,62^. Liberal formal supertree methods enable a well-resolved consensus topology to be estimated from source trees that are incongruent.

Here, the supertree (Fig. S4) was assembled using the matrix representation with parsimony method, an approach that has been validated in methodological comparisons^63^. The supertree was estimated from a sample of 175 source trees published since 2010, each re-analysed from their original source matrices using Bayesian inference and the MK model^64^. Only matrices containing morphological data were used; the source trees relating to the crown-group were constrained to a published topology of extant taxa derived from molecular data. Supertrees generated by the analysis were evaluated using stratigraphic congruence, with the best example being retained for use in analyses. The supertree was dated using the equal method; the dated supertree contained a total of 579 archosauromorph taxa, including 24 extant species. This tree was then trimmed to match the 280 pseudosuchian taxa included in the body size data. This phylogenetic approach was implemented to eliminate as many sources of error as possible. Every effort was taken to incorporate the most recent and comprehensive data from a diversity of morphological, stratigraphic and molecular sources. The best-performing phylogenies are retained at each step. However, like any phylogenetic comparative analysis, the results of the analyses presented here do depend on a correct phylogenetic topology. Therefore if a significant number of the source trees are incorrect, the resulting supertree may not be an accurate framework for phylogenetic comparative methods. This is a limitation that can only be conclusively overcome through further description of pseudosuchian fossils, and continued development of phylogenetic methods. Full details of the supertree analysis are presented in the Supplementary Information.

Phylogenetic models of body size were fitted using BayesTraits version 2^65^. A random walk model was fitted as a null hypothesis and was compared with other models (Ornstein-Uhlenbeck, Kappa, Lambda, Delta, directional trend, variable rates) using Bayes Factors. All analyses were run for two million iterations, and the first 10,000 iterations were deleted as burn-in. The likelihood profiles of each run were plotted to check for a uniform distribution, indicating convergence. Rate scalars returned by the variable rates model were computed using the BayesTraits online post-processing tool, and these were plotted on the phylogenetic tree to illustrate the relative evolutionary rate on each branch. Branch lengths were set to the scalar values returned by the variable rate model, and coloured according to a gradient from blue to red, denoting low and high rates, respectively. The rate scalars were also plotted as a time-series, calculated as averages per 1-million-year time bin, for which the taxa included in each increment were determined using the dated supertree. Bootstrapping was implemented as insurance against the effects of outliers and potential errors introduced by tip dating.

The phylogenetic models return a phylogenetically adjusted mean and root trait estimation, known as alpha (Table 1). An average alpha value was calculated using the values returned by the phylogenetic models that significantly outperformed Brownian motion. This average was weighted using the log Bayes factor of the respective model. For comparison, a simple arithmetic mean was also calculated from the raw data. These mean values are dimensionless, occupying the scale of the first principal component. For the purposes of illustration, the mean values were transformed back into an estimate of skull width in centimetres.

### Time-series analyses

Trees were dated using first and last appearance dates from the Paleobiology Database (pbdb.org). Mean body size and body size variance were represented as time series using the scores of each taxon on principal component 1 as a body size proxy. This is not a straightforward exercise because of fossil preservation heterogeneity and uncertainty about the temporal ranges of taxa, many of which are known from very few specimens. Therefore, we implement three approaches to reconstructing time series of mean body size and body size disparity:

- Empirical fossil ranges. The simplest time-series approach is to bin taxa by the stratigraphic stage in which fossils have been found, a commonly used method^34,35^. This assumes the known stratigraphic range was the true stratigraphic range and does not estimate originations and extinctions before and after the known range.
- Phylogenetic adjustment of missing data. Using the same tip-dated formal supertree used for fitting phylogenetic models, we added ghost ranges in million-year increments (difference in first appearances of sister taxa) to the empirical data in method (a). This approach can only reconstruct ghost ranges and does not estimate beyond known fossil ranges of sister taxa. Further, the method assumes that the topology of the phylogenetic tree is correct; an incorrect topology could significantly change the durations of fossil ghost ranges.
- Addition of ancestral taxa. This method uses stratigraphy and the phylogenetic tree as in method (b), but also estimates traits of hypothetical ancestral taxa, following established methods^66,67^. Ancestral states at each node were estimated using the ACE function in the APE package in R^68^, and their geological age was inferred from the length of the corresponding internal branch of the phylogenetic tree.

A time series may also be biased by outlier taxa, or by inaccuracies in the dating of the phylogenetic tree. To mitigate this, all three versions of each time series were subjected to bootstrapping, in which multiple mean values were calculated from random 50% samples of taxa in each time-bin and repeated over 100 iterations in each case.

The time series of body size, body size variance and evolutionary rate were analysed for correlation with temperature using linear regressions, partitioned into Mesozoic and Cenozoic spans. This temporal split corresponds to the end-Cretaceous mass extinction and an apparent shift in the stability of body sizes. Mesozoic paleotemperatures come from a consensus curve of oxygen isotope data from a range of sources^69^, selected for its comprehensive temporal coverage. Cenozoic paleotemperatures come from a curve estimated from oxygen isotope data derived from benthic foraminifera^70^, which correlates with the less complete terrestrial temperature record^71^ and has been used in previous macroevolutionary analyses of body size^72^. This paleotemperature record is limited to a global average. Similarly, body size data is also limited to a global average, since many species are known from too few specimens for their geographic range to be determined. Therefore the data is not sufficient to incorporate geographic variations in temperature and body size.

### Statistics and reproducibility

Phylogenetic models were fitted using BayesTraits^65^. Statistical analyses were implemented using the R programming language and the APE library^68^. The dataset assembled as part of this study included 280 taxa. Time-series representations were subjected to a sensitivity analysis using 100 randomly sampled bootstrap replicates^73–75^.

### Reporting summary

Further information on research design is available in the Nature Research Reporting Summary linked to this article.

## Supplementary information

Supplementary information

Supplementary Data 1

Reporting Summary

## Data Availability

All data analysed in this study are taken from published sources, including all images of skeletal elements and NEXUS files containing phylogenetic matrices. These published sources are referenced in the Supplementary Information and Supplementary Data 1. All data generated in this study are included in Supplementary Data 1. First-appearance and last-appearance data was accessed from the Paleobiology Database (paleodb.org), and all these data are reproduced in the Supplementary Data 1.
