# Supplementary material for: Environmental drivers of body size evolution in crocodile-line archosaurs
Source: Commun Biol. 2021 Jan 7;4:38. doi: 10.1038/s42003-020-01561-5 (PMC7790829; doi:10.1038/s42003-020-01561-5)
Supplement: Supplementary file 1 — Supplementary information [file 42003_2020_1561_MOESM1_ESM.pdf]

Supplementary Information

Table of contents

Estimation of Phylogeny……………………………………………2
Introduction……………………………………………………2
Limitations of informal supertrees……………………………2
Limitations of supermatrices…………………………………3
Precedent of a formal supertree………………………………3
Materials and methods………………………………………4
Source data collection and preprocessing……………………4
Source tree analysis……………………………………………5
Supertree assembly……………………………………………7
Quality evaluation……………………………………………7
Discussion………………………………………………………8
References………………………………………………………10
Supplementary Table 1……………………………………………15
Traits measured……………………………………………………16

Estimation of Pseudosuchian Phylogeny

Introduction

The analyses presented in this study make use of a phylogenetic tree at multiple stages. A tree is used to estimate the ghost ranges of fossil taxa, the likely trait values of common ancestors and as a framework for analysing evolutionary rates. There are a number of approaches to assembling large phylogenetic trees, each with their own advantages and disadvantages. These include both formal and informal supertrees, and matrix-based approaches that use character data. A formal supertree approach was selected for this study through a process of elimination, since informal supertree and supermatrix approaches present greater systematic and practical issues.

Limitations of informal supertrees

Previous macroevolutionary analyses have frequently relied upon informally assembled supertrees[1,2,3,4,5,6,7]. These are phylogenetic topologies that have been assembled manually, either using a text editor or a phylogenetic tree editor. Typically, researchers use these informal methods to copy sections of topology from multiple previous analyses and unite them into a single tree. Informal supertrees present researchers with a number of advantages. They can be assembled quickly and without specialist software and provide far greater control over the final topology than systematic methods would offer. However, all supertrees, informal or otherwise, share a common drawback that they are dependent on the accuracy of the source trees from which they are estimated. This is especially true of informal trees where topology is copied from older publications, where the data or methodology may be outdated. Informal supertrees are also entirely subjective, and by definition bias analyses in favour of the author's own views. Researchers may attempt to circumvent this problem by performing analyses on multiple versions of a topology. However, the points where topologies may be bisected and recombined are also subjective.

If there is controversy about the evolutionary relationships within a clade, the number of possible informal supertree topologies may become excessive. For example, the positions of several member clades within the Pseudosuchia differ between analyses. The Thalattosuchia have been resolved as a derived clade within the Neosuchia[8,9], a basal sister clade to the Crocodyliformes[10], or an intermediate clade within the Mesoeucrocodylia but outside the Neosuchia[11,12]. There is disagreement over the position of the Peirosauridae and Mahajangasuchidae, with some authors placing them close to the Neosuchia[13] and others the Notosuchia[9]. The Sebecia have been found both as members of the Notosuchia[14] and as a distinct clade[15]. The Phytosauria have been placed inside the Pseudosuchia[16] and outside the Archosauria[17]. Analyses of molecular data position the extant *Gavialis* on a branch with *Tomistoma*, and close to *Crocodylus*[18], while morphological analyses consider *Gavialis* to be the sister taxon to all other living crocodilians. For an analysis to comprehensively consider all of these disagreements would require in excess of forty different topologies. It would be difficult to draw meaningful conclusions from so many trees if they are considered equally likely; it is therefore necessary to develop a consensus of these different viewpoints based on the strongest evidence.

Limitations of supermatrices

Supermatrix approaches avoid many of the subjectivity issues associated with informal supertrees. Supermatrices lack a specific technical definition, however the term is broadly used to describe phylogenetic analysis of a single, comprehensive matrix. A supermatrix of the Pseudosuchia would require in excess of 500 taxa. Estimating such a large phylogenetic tree from a single matrix represents a formidable challenge, either in the sheer number of fossils to be examined and their characters scored, or by the integration of existing matrices. Many data matrices include similar characters that when combined into a single matrix would be redundant. Multiple non-independent characters threaten to over-weight potentially incorrect phylogenetic relationships[19]. Therefore, combining multiple smaller matrices requires careful review of every possible pair of characters to exclude redundant data. The number of checks would approximately equal the square of the sum of the number of characters in all the source matrices. This is not practical for a clade as large as the Pseudosuchia.

Since supermatrix approaches make use of real character data, it can be argued that supermatrix trees will always be superior to supertree approaches. However, this assumption is a substantial one, and under some circumstances may not be the case. Very large morphological character matrices present a significant problem in the accumulation of inapplicable characters (Fig. S1). A comprehensive matrix must inevitably include morphological characters that are not applicable to all its members. For example, a matrix of crocodile-line archosaurs would likely contain characters relating to the morphology of osteoderms, despite osteoderms being absent in some members. Therefore, as matrices become larger, the percentage of the matrix dedicated to inapplicable characters increases. When combined with the incompleteness inherent to fossil data, large matrices of fossil taxa may accrue large quantities of missing data. If a logistic curve is fitted through a bivariate space of matrix completeness against the number of taxa in a sample of pseudosuchian data matrices (Fig. S2), it predicts the completeness of very large matrices will be low. A data matrix of over 500 taxa, as would be required to comprehensively represent the Pseudosuchia, may be expected to contain at least 50% missing data. Low matrix completeness may be expected to have an impact on the efficacy of phylogenetic analyses. Therefore, it is not reasonable to assume that the time invested in building a very large supermatrix will be rewarded with a high-quality phylogenetic analysis. The practicalities and quality issues surrounding very large supermatrices may explain why they have not seen more widespread use in studies of fossil macroevolution.

Precedent for a formal supertree

The analysis presented here uses a formal supertree approach. Formal supertrees combine smaller source topologies into a single larger supertree, similar to informal supertree approaches. However, formal supertree methods use a systematic numerical procedure and phylogenetic analysis to build the tree. The supertree analysis presented in this study implements the Matrix Representation Parsimony (MRP) method. The MRP method is a well-established approach and has been applied in many examples using fossil data, including early tetrapods[20], dinosaurs[21,22], and crocodyliformes[23,24]. Formal supertree methods generally fall into one of two classes, termed liberal and conservative approaches. Conservative approaches handle incongruences between source trees by presenting them as unresolved nodes in the final topology[25]. Conservative supertree

approaches are not suitable for this study, since the finished tree must be fully resolved to be useful for comparative phylogenetic methods. The MRP method is an example of a liberal supertree approach, where incongruences between source trees are resolved democratically, with the better-supported topology being retained in the final supertree. The MRP method is a pragmatic choice, since it can be implemented using readily available software without consuming excessive computer processing power.

Formal supertree methods have been subject to controversy and are not without issues. Studies sceptical of supertrees, such as Gatesy *et al*., have concluded that these issues are insurmountable and that supertree methods should be avoided altogether[26]. A rebuttal by Bininda-Emonds *et al*. concluded that these problems could be mitigated through careful source tree selection protocols and stated that supertrees are a necessity due to the inherent impracticality of supermatrices[27]. Like the more widely used informal supertrees, formal supertrees can only be as accurate as the source trees from which they are estimated. In addition, if source trees are not selected carefully, formal supertrees may become biased towards the opinions of the most prolific authors. The MRP method in particular may be biased towards asymmetric trees[28]. This does not put it at a disadvantage compared to other formal supertree methods, which may be biased towards other tree shapes 28. Akanni *et al.* found that the MRP method outperformed other methods in its representation of source trees[29]. It must also be considered that any systematic bias introduced by formal supertree methods may compare favourably with subjective bias introduced by the widely used informal approach. This study responds to concerns surrounding the MRP method through re-analysis of carefully selected source trees, and thorough quality evaluation of the finished tree. The topology of the finished supertree can be compared with previously published phylogenetic analyses, to ensure that the final topology is realistic. It is unlikely that biases may be eliminated entirely, however with careful implementation and quality control the standard of a formal supertree can be raised to at least that of the informal trees used in previous macroevolutionary studies.

Materials and methods

Source data collection & preprocessing

Supertrees were assembled from a dataset of source trees re-analysed from published data matrices. Reanalysing matrices enables each source tree to be raised to an equal standard, and any phylogenetic methods used in the source publication to be updated. The literature was searched for research articles in peer-reviewed academic journals featuring systematic analyses of phylogeny, with at least one matrix of character data included in the supplementary information. If multiple character matrices were included in a source publication, they would each be included in the source tree database. Matrices were included if they included at least 5 pseudosuchian taxa. Including non-pseudosuchian taxa gives a foundation of additional data to support the root of the tree. In addition, the inclusion of more basal archosauromorph taxa tests the membership of the Pseudosuchia in uncertain clades such as the Phytosauria. The sampling window for source analyses was limited to those published since 2010. This was picked as a starting date so that only the most recent and up-to-date analyses were included. Limiting the sampling window in this manner will help to mitigate the effects of author bias, since more prolific authors make their contributions over a longer period. Therefore using a

short sampling window restricts the contributions of prolific authors to only their most recent publications. However, author bias is something of an intractable issue among archosauromorphs, since even less prolific authors commonly adapt matrices published by their more prolific colleagues. Starting the sampling window in 2010 presents an additional advantage, since it pre-dates previous large-scale reviews of archosauromorph phylogeny[13,23]. This approach reduces the number of source publications not included in a meta-analysis, thereby reducing the possibility that major breakthroughs have been overlooked. This approach to data collection recovered a total of 175 source publications.

The taxonomy of the source matrices was standardized according to a set of accepted names download from the Paleobiology Database (pbdb.org, supplementary information). Higher-rank terminal taxa were raised to species level resolution across all matrices. Increasing phylogenetic resolution in this manner can be subjective; the supertree by Bronzati *et al*. inserted polytomies including all members of each higher rank taxon[13]. However, this approach requires a prior understanding of the complete phylogeny. Therefore, this may either defeat the purpose of building a large phylogeny, or worse bias the result towards the author's own opinion. To mitigate these concerns, higher-rank terminal taxa in the source analyses recovered for this study were replaced by a single member species, picked at random. While this approach demands confidence that the chosen species is indeed a member of the higher-rank taxon it represents, it is less subjective than assigning multiple member species to a polytomy. Rows in source matrices pertaining to undescribed specimens were left unaltered in the source matrices; this additional data is retained so each source analysis has the greatest possible quantity of data available. Undescribed specimens would then be removed for the meta-analysis at a later stage, so the finished tree contains only valid taxa at species resolution.

Source tree analysis

In recent years there has been debate over the optimum methodology for analysing morphological data matrices. Some authors have argued that maximum parsimony is ill-equipped to deal with homoplasy and character state reversions and may tend to force a maximum resolution even when support for a given node may be modest[30]. There have been a number of studies where the authors suggest Bayesian methods may outperform parsimony even with very simple models such as the MK model[30,31,32]. Critics of these analyses have argued that the MK model does not reflect morphological evolution in a realistic way[33,34]. In particular, the MK model assumes that the distribution of character states and the rate of character substitutions are constant. The MK model only seeks to minimise rate shifts within the tree. The debate over which method is superior remains unresolved. To take this controversy into account, the source analyses for this study were each analysed twice: once using maximum parsimony, and once using Bayesian inference and the MK model. Parsimony analysis of each source matrix was implemented using a new technology search in TNT[35] set to 50 hits (Fig. S3a). All four tree-search algorithms were employed. The trees returned by each parsimony analysis were combined into a strangle source topology using a strict consensus approach. The strict consensus was used as a more conservative estimate than a majority-rule consensus (Fig. S3b).

Analysis of the source matrices using Bayesian inference was implemented using MrBayes[36]. Rates were drawn from a gamma distribution, with the MK model[37] used as

the optimization criteria (Fig. S3a). Each analysis was performed using two MCMC chains, each set to a total 500 thousand generations and sampled every 500 trees. Each analysis was provided with a random tree taken from the corresponding parsimony analysis as a starting tree. Testing for convergence was performed using effective sample size implemented in Tracer[38]. An analysis was considered to have achieved convergence if it returned an average effective sample size of 200 or more across the four estimation methods implemented in Tracer. Analyses that did not achieve an average effective sample size of 200 were deleted from the dataset. To ensure consistency between the parsimony and Bayesian approaches, the corresponding analyses were also deleted from the parsimony source tree sample. The first 25% of the MCMC samples were deleted as burn-in. The remaining post-convergence sample was combined into a single topology using a majority-rule consensus. Due to the nature of Bayesian inference, a strict consensus of these samples would return no topology. Therefore, trees generated by the MCMC chains post convergence were summarized using a majority rule-consensus (Fig. S3b). The decision to use a majority-rule consensus was taken in the light of the findings of O'Reilly and Donoghue, which concluded that majority-rule consensus trees are less prone to finding incorrect or poorly supported trees than maximum clade credibility (MCC) consensus trees [39].

As noted previously, the phylogenies recovered by previous analyses of the crown-group Crocodylia are highly incongruent, depending on what type of data is used. Phylogenetic topologies estimated from morphological data position the genus *Gavialis gangeticus* as the sister taxon to all other extant Crocodylia. The false gharial, *Tomistoma schlegelii*, is found to be more closely affiliated with a monophyletic clade of true crocodiles. However, phylogenetic topologies estimated from molecular data find *Gavialis gangeticus* and *Tomistoma schlegelii* to form a sister clade to the extant Crocodylia. A supertree of the total-group Pseudosuchia must take these incongruences into account. There are good reasons to favour phylogenetic analyses of molecular data over those of morphology. Morphological characters of any given taxa are subject to selection pressure, and therefore cannot be assumed to be independent from one another due to coevolution and modularity. This places phylogenetic analysis of morphological characters at a disadvantage compared to analyses of molecular data. Non-coding DNA has no manifestation in a phenotype; therefore, variations are not subject to selection pressure. Coding DNA sequences will be subject to selection pressure, but to a far lesser extent than morphology. Multiple different codon triplets may encode a given amino acid in a protein. Therefore, coding DNA may sustain a considerable degree of interspecies variation without expressing any change in function for selection to act upon. Phylogenetic analyses of morphology are further hampered due to taxon completeness, and homoplastic characters make resolving topology difficult. Molecular data matrices can be expected to have greater completeness than morphological data from fossils. The decay of the fossil record results in continual loss of data over time. DNA is common to all organisms, unlike morphological characters, which may be limited to certain groups. Further, molecular data presents an additional advantage in the quantity of character states attributable to a single taxon. Diagnosing sufficient morphology to distinguish taxa can be difficult, especially in examples where morphological disparity is low. Molecular data can include orders of magnitude more character states, regardless of morphological disparity in the sample taxon. This in turn offers greater confidence in the topologies thus inferred. For the purposes of this study, phylogenetic analyses of molecular data are assumed to be more accurate than those of morphological data. The results of previous molecular analyses were incorporated into

the supertree using topological constraints. A topological constraint could easily be applied to each supertree assembly. However, in a supertree tree assembled in this manner, extinct taxa would be expected to inherit the topology estimated in an unconstrained analysis. This may be inaccurate, especially in the case of source trees inferred using Bayesian inference, where the rate and timing of nodes is taken in to account. Therefore, instead a topological constraint was applied to each source analysis and the supertree assembly left unconstrained. The constraint implemented was a topology of extant taxa taken from Lee and Yates[40]. This topology was selected for a number of reasons: it is very recent, comprehensive, and has been assembled in a Bayesian framework using molecular data.

Supertree assembly

Supertrees were assembled from each set of source trees using the matrix representation parsimony method (Fig. S3d). The MRP method decomposes source trees into a matrix of virtual characters. For every given source tree, a virtual character is defined for each node. The descendants of a given node are all scored as 1, and the remaining source tree taxa that do not descend from that given node are scored as 0. Taxa to be included in the supertree that are missing from a given source tree are scored as unknown, with a question mark. The root nodes of each source tree are omitted from the scoring process, since all the taxa in that source tree will be descended from it. This would result in an invariant virtual character that is not useful for phylogenetic analysis. The finished MRP matrix will contain as many virtual characters as the sum total of the number of nodes in all the source trees, minus the number of trees. The completed virtual character matrix is then analysed using maximum parsimony. Virtual character data is well-suited to parsimony analysis, since by definition virtual characters cannot undergo state reversion or emerge convergently in multiple taxa. This interpretation of the MRP method is that defined by Baum and Regan[41,42]. It differs from that of Purvis, which defines a virtual character as present in all descendants of a given node, but absent only in the sister taxon to that node, with the remaining tips on the source tree being classified as unknown[43]. The Purvis method has a tendency to return less well-resolved trees than the Baum and Regan method[43]. The Purvis method was devised in an attempt to limit the data redundancy in MRP data, but Ronquist concluded that the data removed by the Purvis method was non-redundant[44]. Undescribed specimens and invalid taxa were retained during the scoring process, in order to maximize the available virtual character data. However, once the character scoring was completed, these rows were deleted from the matrix. This was step was taken to reduce the size of the tree space to be searched and ensuring that only valid taxa remain in the completed supertree. The MRP matrix was furnished with an all-zero pseudo-outgroup, following the methodology of Bronzati *et al.*[23]. The completed MRP matrix was analysed using a new technology search in TNT set to 100 hits, with all four search algorithms.

Quality evaluation

Both parsimony-based and Bayesian inference-based MRP analyses returned multiple trees. The MRP analysis using parsimony-derived source trees found four equally parsimonious trees. The analysis using Bayesian-inference derived source trees identified two trees. The efficacy of each of these trees needed to be compared systematically in order to identify one fully resolved supertree that outperforms the

other five. One approach would be to calculate the MK likelihood or parsimony score of each tree when compared to a supermatrix of Psuedosuchian morphological data. However, as discussed previously such a matrix would be impractical to assemble and could introduce quality issues. The analysis of Allen *et al.* evaluated informal supertrees using supertree likelihoods[1], as defined by Steel and Rodrigo (2008)[45]. This approach calculates the supertree likelihood from the Robinson-Foulds distance between the supertree and each of its source trees. This approach is therefore not appropriate for this analysis because there are two source tree samples, one estimated using parsimony and the other Bayesian inference. It isn't possible to say with certainty which of these two source tree samples is the more accurate.

The supertrees returned by the two MRP analyses were instead evaluated using stratigraphic correlation (table S1). The best tree is assumed to be that which explains the distribution of fossils through time. Stratigraphic correlation was implemented using the Strap package in R[46]. First- and last-appearance dates for each taxon in the tree were taken from the Paleobiology Database (pbdb.org). If a taxon profile did not include a first- or last-appearance date, then this taxon was deleted from the correlation analysis. The correlation analysis was implemented using the equal tree dating method, with 10 resamples. The analysis returned four metrics of stratigraphic correlation: the Stratigraphic Consistency Index (SCI), Relative Completeness Index (RCI), the Gap Excess Ratio (GER) and the Manhatten Stratigraphic Measure (MSM). This analysis identified a supertree estimated from Bayesian source trees that showed greater stratigraphic correlation than the other supertrees recovered.

The most stratigraphically congruent tree was pruned to include only taxa for which relative body size estimates were available (Fig. S4). The pruned supertree was evaluated through comparison with published phylogenetic trees to check that the topology was, albeit subjectively, within the bounds of plausibility (see discussion). The pruned supertree was tip-dated using the equal method implemented in Strap package in R, with a minimum branch length of 1 million years. This ensured that the dated tree was without zero-length branches, and therefore compatible with software tools to implement phylogenetic comparative methods. Other methods, such as the Cal3 method, may produce more accurate dating, but relies upon a prior estimate of evolution and extinction rates, which is difficult to estimate when taxa are known from very few specimens. The tip-dating procedure in this study used the same stratigraphic data as the stratigraphic correlation analysis, downloaded from the PaleoBiology Database. The pruned and dated tree was retained for use in comparative phylogenetic analyses.

Discussion

The supertree identifies the Phytosauria as a monophyletic group within Pseudosuchia, closer to extant crocodilians than to Avemetatarsalia. A monophyletic clade of ornithosuchid genera lies crownward of the phytosaurs, including *Ornithosuchus*, *Riojasuchus* and *Venaticosuchus*. These observations concur with the findings of Ezcurra *et al.*[16]. Crownward of the Ornithosuchidae lies a monophyletic Aetosauria, broadly divided into two monophyletic subclades, corresponding to the Typothoracisinae and Desmatosuchinae. Still further crownward of the Aetosauria is a monophyletic Poposauroidea, subdivided into two smaller clades corresponding to the Shuvosauridae and the Ctenosauriscidae. The supertree finds *Qianosuchus* to be outside either of these clades. This topology is comparable with that recovered by Nesbitt *et al.*[47].

Taxa described as either sphenosuchian or protosuchian are found to form paraphyletic grades of basal crocodylomorphs, rather than monophyletic groups. This concurs with Nesbitt *et al.*[47]. The supertree finds the Mesoeucrocodylia to be divided approximately into two major branches, corresponding to the Notosuchia and Neosuchia. *Anatosuchus* is found to be the sister taxon to all other Notosuchia. A paraphyletic grade of basal Notosuchians precedes a monophyletic Notosuchidae. The Notosuchidae is found to be the sister clade to the common ancestor of the Baurusuchidae and the Sebecidae. This is consistent with published analyses including Adams et al. and Pol et al.[48,49]. However, this does conflict with a previous analysis by Larsson and Sues, who concluded that the Baurusuchidae and Sebecidae lie closer to the Neosuchia than the Notosuchia[15].

Neither analysis found the Peirosauridae or Mahajangasuchidae to be affiliated with the Notosuchia, instead placing them crownward of the Notosuchia as a sister clade to the Neosuchia. This is unlike the topology estimated by Turner and Sertich, Pol et al. and Turner, who concluded the Peirosauridae to be closer to the Notosuchia[9,14,49], but congruent with the conclusions of Sereno and Larsson, Riff and Kellner and Wilberg et al.[13,15,50].

Both metatrees find a monophyletic clade of atoposaurids as basal stem Neosuchians, including *Theriosuchus* and *Sabresuchus*. This agrees approximately with the findings of Tennant *et al.*[15]. *Atoposaurus* and *Alligatorellus* are found to be outside this clade, together forming a paraphyletic grade of basal Neosuchians. This reflects a broader uncertainty about the phylogeny of these taxa. Tennant *et al.* recover a diversity of different phylogenetic arrangements depending on what methods are used.

The supertree finds the Tethysuchia and Thalattosuchia to form sister clades within the Neosuchia. They are distinct from a second major clade of Neosuchia, comprising of the Goniopholididae, Paralligatoridae and the Eusuchia. This topology is in overall agreement with that of Turner[14], but contrasts with that of Adams *et al.* and Andrade *et al.*, who placed the Goniopholididae closer to the Tethysuchia and Thalattosuchia[48,52]. The positioning of the Thalattosuchia has been a matter of considerable controversy. They have been resolved as a derived clade within the Neosuchia[14,52], a basal sister clade to the Crocodyliformes[10], or an intermediate clade within the Mesoeucrocodylia but outside the Neosuchia [11,12]. The supertree finds the Tethysuchia to form two subclades corresponding to the Elosuchia and the Dyrosauridae. This is concordant with Andrade *et al.*[52]. The supertree identifies a monophyletic family of basal Thalattosuchia corresponding to the Teleosauridae, and a derived family corresponding to the Metriorhynchidae. Some previous analyses have found the Teleosauridae to be paraphyletic, however monophyletic interpretations have arisen more recently [14,53]. The supertree finds the Metriorhynchidae to partition into two smaller monophyletic groups, the Metriorhynchinae and the Geosaurinae. This topology agrees with the majority of recent analyses[14,48,52,53]. The topology of the Goniopholididae shows overall similarity with Turner, with *Calsoyasuchus* and *Sunosuchus* emerging in a basal clade separate from later goniopholidid taxa[14].

The topology of the crown-group is dictated in large part by the topological constraints placed upon the source trees. This result demonstrates that the MRP method is capable of producing high-quality supertrees, since the constraint was applied to the source trees but not the supertree itself. Despite this, the constrained topology has been retained by the supertree analysis, suggesting that the source trees have been integrated correctly. Gavialid and tomistomine taxa form a single clade, the sister taxon to the true crocodiles. Analysis by Lee and Yates concluded that Eosuchus and Thoracosaurus form

a clade of stem-group Eusuchia[14]. This relationship is not sustained by the supertree, instead resolving these taxa as members of the Gavialidae, comparable with analyses by Brochu and Wu and Brinkman[54,55]. The Mekosuchinae emerge as the sister clade to the crown-group crocodiles. The Osteolaeminae form a monophyletic group separate from *Mecistops* and *Crocodylus*. *Crocodylus* consistently falls into two broad subgenera, separating African and American species from those in Asia and Australia. The topology of the true crocodiles in the supertree is comparable with that recovered by Brochu and Storrs[56]. The supertree finds *Leidyosuchus*, *Deinosuchus* and *Diplocynodon* to form a grade of basal alligatorids. Alligators and caimans form sister clades, with the giant *Purussaurus* and bizarre platyrostrine *Mourasuchus* lying within the caiman crown-group. This is comparable with Brochu and Lee and Yates[14,57].

The structure of the most stratigraphically congruent supertree shows general agreement with previous phylogenetic analyses. While the supertree topology favours some published interpretations of pseudosuchian phylogeny over others, it cannot reasonably be described as novel. The supertree has also retained the constraints applied to the source topologies. These observations suggest that the formal supertree approach has integrated the source topologies to a standard equal to or surpassing that of informal trees used previously. In addition, this formal supertree incorporates both molecular and probabilistic approaches to phylogeny. The supertree was therefore deemed of a sufficiently high standard for use in phylogenetic comparative methods.

***References**:*

1. Allen, B. J., Stubbs, T. L., Benton, M. J., Puttick, M, N. Archosauromorph extinction selectivity during the Triassic–Jurassic mass extinction. *Palaeontology,* **62**, 211-224 (2019).

2. Brocklehurst, R. J., Schachner, E. R. & Sellers W I. Vertebral morphometrics and lung structure in non-avian dinosaurs. *Roy. Soc. open. sci.* **5**, 180983 (2018).

3. Button, D. J., Lloyd, G. T., Ezcurra, M. D. & Butler, R. J. Mass extinctions drove increased global faunal cosmopolitanism on the supercontinent Pangaea. *Nat. Commun.* **8**, 733 (2017).

4. Dunne, E. M., Close, R. A., Button, D. J., Brocklehurst N., Cashmore, D. D., Lloyd, G. T. & Butler, R. J. Diversity change during the rise of tetrapods and the impact of the 'Carboniferous rainforest collapse'. *Proc. R. Soc. B.* **285**, 20172730 (2018).

5. Famoso, N. A, Davis, E. B., Feranec, R. S., Hopkins, S. S. & Price, S. A. Are hypsodonty and occlusal enamel complexity evolutionarily correlated in ungulates? *J. Mamm. Evol.* **23**, 43-47 (2016).

6. Foth, C. & Joyce, W. G. Slow and steady: the evolution of cranial disparity in fossil and recent turtles. *Proc. R. Soc. B.* **283**, 20161881 (2016).

7. Hedrick, B. P., Manning, P. L., Lynch, E. R., Cordero, S. A. & Dodson, P. The geometry of taking flight: limb morphometrics in Mesozoic theropods. *J. Morphol.* **276**, 152-166 (2015).

8. Andrade, M. D., Edmonds, R., Benton, M. J. & Schouten, R. A new Berriasian species of *Goniopholis* (Mesoeucrocodylia, Neosuchia) from England, and a review of the genus. *Zool. J. Linn. Soc.* **163**, 66-108 (2011).

9. Turner, A. H. A review of *Shamosuchus* and *Paralligator* (Crocodyliformes, Neosuchia) from the Cretaceous of Asia. *PLoS One* **10**, e0118116 (2015).

10. Jouve, S. The skull of *Teleosaurus cadomensis* (Crocodylomorpha; Thalattosuchia), and phylogenetic analysis of Thalattosuchia*. J. Vert. Paleontol.* **29**, 88-102 (2009).

11. Sereno, P. C. & Larsson, H. C. Cretaceous Crocodyliforms from the Sahara. *ZooKeys* **28**, 1-143 (2009).

12. Young, M. T. & Andrade, M. B. What is Geosaurus? Redescription of *Geosaurus giganteus* (Thalattosuchia: Metriorhynchidae) from the Upper Jurassic of Bayern, German. *Zool. J. Linn. Soc.* **157**, 551-585 (2009).

13. Wilberg, E. W. A new metriorhynchoid (Crocodylomorpha, Thalattosuchia) from the Middle Jurassic of Oregon and the evolutionary timing of marine adaptations in thalattosuchian crocodylomorphs. *J. Vert. Paleontol.* **35**, e902846 (2015).

14. Turner, A. H. & Sertich, J. W. Phylogenetic history of *Simosuchus clarki* (Crocodyliformes: Notosuchia) from the Late Cretaceous of Madagascar. *J. Vert. Paleontol.* **30**, 177-236 (2010).

15. Larsson, H. C. E. & Sues, H. D. Cranial osteology and phylogenetic relationships of *Hamadosuchus rebouli* (Crocodyliformes: Mesoeucrocodylia) from the Cretaceous of Morocco. *Zool. J. Linn. Soc.* **149**, 533-567 (2007).

16. Ezcurra, M. D. The phylogenetic relationships of basal archosauromorphs, with an emphasis on the systematics of proterosuchian archosauriforms. *PeerJ* e1778 (2016).

17. Nesbitt, S. J., Desojo, J., Irmis, R. B. Anatomy, Phylogeny and Palaeobiology of Early Archosaurs and their Kin, Geological Society Special publication (2013).

18. Oaks, J. R. A time-calibrated species tree of the Crocodylia reveals a recent radiation of the true crocodiles. *Evolution* **65**, 3285-3297 (2011).

19. O'Keefe, R. R. Wagner, P J. Inferring and testing hypotheses of cladistic character dependence by using character compatibility. *Syst. Bio.* **50**, 657- 675 (2001).

20. Ruta, M., Jeffery, J. E. & Coates, M. I. A supertree of early tetrapods. *Proc. R. Soc. B.* **270**, 2507-2516 (2003).

21. Lloyd, G. T., Davis, K. E., Pisani, D., Tarver, J. E., Ruta, M., Sakamoto, M., Hone, D. W. E., Jennings R. & Benton M. J. Dinosaurs and the Cretaceous Terrestrial Revolution. *Proc. R. Soc. B.* **275**, 2483-2490 (2008).

22. Lloyd, G. T., Bapst, D. W., Friedman, M. & Davis, K. E. Probabilistic divergence time estimation without branch lengths: dating the origins of dinosaurs, avian flight and grown birds. *Biol. Lett.* **12**, 20160609 (2016).

23. Bronzati, M., Montefeltro, F. C. & Langer, M. C., A species-level supertree of Crocodyliformes. *Historical Biology* **24**, 598-606 (2012).

24. Bronzati, M., Montefeltro, F. C. & Langer, M. C. Diversification events and the effects of mass extinctions on Crocodyliformes evolutionary history. *Roy. Soc. open. sci.* **2**, 140385 (2015).

25. McMorris, F. R. & Wilkinson, M. Conservative supertrees. *Syt. Bio.* **60**, 232-238 (2011).

26. Gatesy, J., Baker, R. H. & Hayashi, C. Inconsistencies in arguments for the supertree approach: supermatrices versus supertrees of Crocodylia. *Syt. Bio.* **53**, 342-355 (2004).

27. Bininda-Edmons, O. R. P., Jones, K. E., Price, S. A., Grenyr, R., Cardillo, M., Habib, M., Purvis, A. & Gittleman, J. L. Supertrees are a necessary not-so- evil: a comment on Gatesy et al. *Syt. Bio.* **52**, 724-729 (2003).

28. Wilkinson, M., Cotton, J. A., Creevey, C., Eulenstein, O., Harris, S. R., Lapointe, F.-J., Levasseur, C., Mcinerney, J. O., Pisani, D. & Thorley, J. The shape of supertrees to come: tree shape related properties of fourteen supertree methods. *Syt. Bio.* **54**, 419-431 (2005).

29. Akanni, W. A., Wilkinson, M., Creevey, C. J., Foster, P. G. & Pisani, D. Implementing and testing Bayesian and maximum-likelihood supertree methods in phylogenetics. *Roy. Soc. open. sci.* e140436 (2015).

30. O'Reilly, J. E., Puttick, M. N., Parry, L. A., Tanner, A., Tarver, J. E., Fleming, J., Pisani, D. & Donoghue, P. C. J. Bayesian methods outperform parsimony but at the expense of precision in the estimation of phylogeny from discrete morphological data. *Biol. Lett.* **12**, 20160081 (2016).

31. Puttick, M. N., O'Reilly, J. E. Tanner, A., Fleming, J. F., Clark, J., Holloway, L., Lozano-Fernandez, J. F., Parry, L. A., Tarver, J. E., Pisani, D. & Donoghue, P. C. Uncertain-tree: discriminating among competing approaches to the phylogenetic analysis of phenotype data. *Proc. R. Soc. B.* **284**, 20162290 (2017).

32. O'Reilly, J. E., Pisani, D. & Donoghue, P. C. J. Probabilistic methods that surpass parsimony when assessing clade support in phylogenetic analyses of discrete morphological data. *Palaeontology* **61**, 105-118 (2017).

33. Goloboff, P. A., Galvis, A. T. & Arias, J. S. Parsimony and model-based phylogenetic methods for morphological data: Comments on O'Reilly et al. *Palaeontology* **61**, 625-630 (2018).

34. Goloboff, P. A., Torres, A. & Salvador, A. J. Weighted parsimony outperforms other methods of phylogenetic inference under models appropriate for morphology. *Cladistics* **34**, 407-437 (2017).

35. Goloboff, P. A. & Catalano, S. A. TNT version 1.5, including a full implementation of phylogenetic morphometrics. *Cladistics* **32**, 221-238 (2016).

36. Ronquist, F. & Huelsenbeck, J. P. MrBayes 3: Bayesian phylogenetic inference under mixed models. *Bionformatics* **19**, 1572-1574 (2003).

37. Lewis, P. O. A likelihood approach to estimating phylogeny from discrete morphological character data. *Syst. Bio.* **50**, 913-925 (2001).

38. Rambaut, A. Suchard, M. A., Xie W. & Drummond A. J. MCMC Trace Analysis Tool. Tracer 1.6.1 (2003).

39. O'Reilly, J. E. & Donoghue, P. C. The efficacy of consensus tree methods for summarizing phylogenetic relationships from a posterior sample of trees estimated from morphological data. *Syst. Bio,* **67**, 354-362 (2017).

40. Lee, M. S. Y. & Yates, A. M. Tip-dating and homoplasy: reconciling the shallow molecular divergences of modern gharials with their long fossil record. *Proc. R. Soc. B.* **285**, 20181071 (2018).

41. Baum, B. R. Combining trees as a way of combining data sets for phylogenetic inference, and the desirability of combining gene trees. *Taxon* **41**, 3-10 (1992).

42. Regan, M. A. Phylogenetic inference based on matrix representation of trees. *Mol. Phylogenetics Evol.* **1**, 53-58 (1992).

43. Purvis, A. A modification to Baum and Regan's method fro combining phylogenetic trees. *Syst. Bio.* **44**, 251-255 (1995).

44. Ronquist, F. Matrix representation of trees, redundancy and weighting. *Syst. Bio.* **45**, 247-253 (1996).

45. Steel, M., Rodrigo, A. Maximum likelihood supertrees. *Syst. Bio.* **57**, 243-250 (2008).

46. Bell, M. A. & Lloyd, G. T. Strap: an R package for plotting phylogenies against stratigraphy and assessing their stratigraphic congruence. *Palaeontology* **58**, 379-389 (2015).

47. Nesbitt, S. J., Sidor, C. A., Angielczyk, K. D., Smith, R. M. H. & Tsuji, L. A. A new archosaur from the Manda beds (Anisian, Middle Triassic) of southern Tanzania

and its implications for character state optimizations at Archosauria and Pseudosuchia. *J. Vert. Paleontol.* **34**, 1357-1382 (2014).

48. Adams, T. L. A small crocodyliform from the Lower Cretaceous (late Aptian) of central Texas and its systematic relationship to the evolution of Eusuchia. *J. Vert. Paleontol.* **88**, 1031-1049 (2014).

49. Pol, D., Nascimento, P. M., Carvalho, A. B., Riccomini, C., Pires-Domingues, R. A. & Zaher, H. A new notosuchian from the Late Cretaceous of Brazil and the phylogeny of advanced notosuchians. *PLOS ONE* **9**, e93105 (2014).

50. Riff, D & Kellner, A. W. A. Baurusuchid crocodyliforms as theropod mimics: clues from the skull and appendicular morphology of *Stratiotosuchus maxhechti* (Upper Cretaceous of Brazil). *Zool. J. Linn. Soc.* **163**, 37-56 (2011).

51. Tennant, J. P., Mannion, P. D., Upchurch, P. Evolutionary relationships and systematics of Atoposauridae (Crocodylomorpha: Neosuchia): implications for the rise of Eusuchia. *Zool. J. Linn. Soc.* **177**, 854-936 (2016).

52. Andrade, M. D., Edmonds, R., Benton, M. J. & Schouten, R. A new Berriasian species of *Goniopholis* (Mesoeucrocodylia, Neosuchia) from England, and a review of the genus. *Zool. J. Linn. Soc.* **163**, 66-108 (2011).

53. Young, M. T., Brusatte, S. L., Andrade, M. B., Desojo, J. B., Beatty, B. L., Steel, L., Fernandez, M. S., Sakamoto, M., Ruiz-Omenaca, J. I. & Schoch, R. R. The cranial osteology and feeding ecology of the metriorhynchid crocodylomorph genera *Dakosaurus* and *Plesiosuchu*s from the Late Jurassic of Europe. *PLOS ONE* **7**, e44895 (2012).

54. Brochu, C. A. Morphology, fossils, divergence timing, and the phylogenetic relationships of *Gavialis*. *Syst. Bio.* **46**, 479-522 (2013).

55. Wu, X. C. & Brinkman, D. B. A new crocodylian (Eusuchia) from the uppermost Cretaceous of Alberta, Canada. *Can J. Earth Sci.* **52**, 590-607 (2015).

56. Brochu, C. A., & Storrs, G. W. A giant crocodile from the Plio-Pleistocene of Kenya, the phylogenetic relationships of Neogene African crocodylines, and the antiquity of *Crocodylus* in Africa. *J. Vert. Paleontol.* **32**, 587- 602 (2012).

57. Brochu, C. A. Phylogenetic relationships of *Necrosuchus ionensis*, Simpson, 1937 and the early history of caimanines. *Zool. J. Linn. Soc.* **163**, 228-256 (2011).

	Tree No.	SCI	RCI	GER	MSM
Parsimony-derived source trees	1	0.44791667	-1893.1427	0.62597257	0.00964163
	2	0.44097222	-1907.2858	0.62329267	0.0095737
	3	0.453125	-2116.899	0.58357431	0.00866848
	4	0.44965278	-2152.9338	0.57674629	0.00852983
Bayesian-derived source trees	1 *	0.453125	*-971.81663*	*0.80054917*	*0.01792951*
	2	*0.46354167*	-975.02973	0.79994034	0.01787592

Supplementary Table 1: Stratigraphic correlation metrics of the supertrees returned by the matrix representation with parsimony analyses. Best supported trees for each metric are indicated in italics. SCI refers to the Stratigraphic Correlation Index, RCI to the Relative Completeness Index, GER the Gap Excess Ratio and MSM to the Manhatten Stratigraphic Measure. The tree indicated with an asterisk (*) is the one carried forward for use in macroevolutionary analyses. Stratigraphic correlations were implemented using the Strap package in R.

Traits measured

The principal components analysis in this study used as input a matrix of 21 characters:

1. **Skull width**: The width of the skull at the widest point of the quadrate bones.
2. **Skull length**: The length of the skull from the occipital to the tip of the snout.
3. **Interorbital**: The distance between the orbits at their narrowest point.
4. **Orbit length**: The length of the orbit along the anteoposterior axis.
5. **Snout width**: The width of the skull at the margin between the maxilla and premaxilla.
6. **Snout width:** The width of the skull at the margin between the maxilla and the jugal.
7. **Nasal length**: The length of the nasal along the anteoposterior axis.
8. **Post-orbital fenestrae width**: The width of the post-orbital fenestrae along the mediolateral axis.
9. **Post-orbital fenestrae length**: The length of the post-orbital fenestrae along the anteoposterior axis.
10. **Frontal – supraocciptal**: The length from the supraoccipital to the anterior-most point of the frontal bone.
11. **Skull depth**: the depth of the skull at the margin between prefrontal and nasal bones.
12. **Skull depth**: the depth of the skull at the margin between maxilla and premaxilla bones.
13. **Mandibille length**: Total length of the lower jaw along the anteoposterior axis.
14. **Mandabille depth**: The depth of the mandibille at the margin between the dentiary and the surrangular.
15. **Mandibille width:** The width of the mandibille at the articular bones.
16. **Dentiary suture**: The length of the suture between left and right dentiaries along the anteroposterior axis
17. **Mandibille width**: The width of the lower jaw at the margin between the angular and the dentiary.
18. **Femur head width**: The width of the femur head along the mediolateral axis.
19. **Femur length**: The length of the femur.
20. **Humerus length**: The length of the humerus.
21. **Humeral head width**: The width of the humeral head along the mediolateral axis.

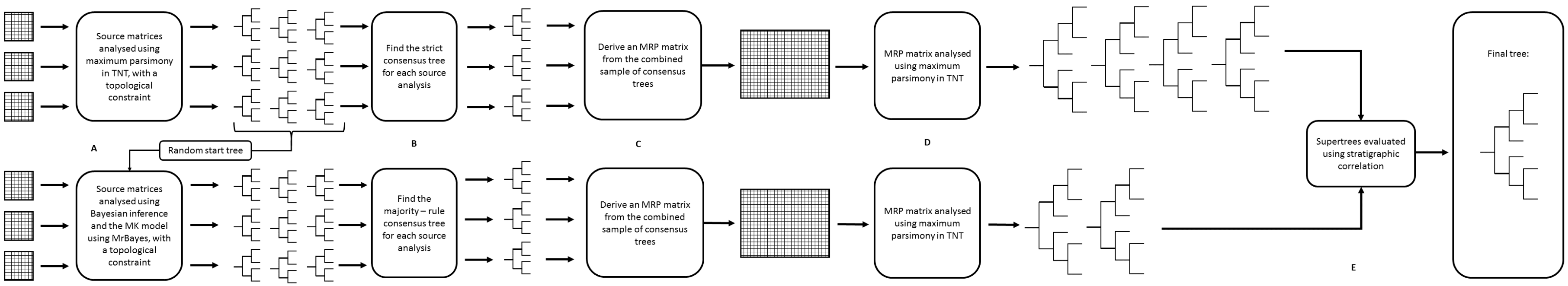

Fig. S1: Stylised representation of the supertree assembly pipeline. Source matrices were analysed using both maximum parsimony and Bayesian inference (*a*). The source analyses were constrained to fit a molecular topology of extant taxa. Each source analysis was aggregated into a single source tree using consensus trees (*b*). Separate virtual character matrices were derived from parsimony- and Bayesian-derived source trees (*c*). These matrix representations were both analysed using maximum parsimony (*d*). The supertrees returned by these analyses were then evaluated using stratigraphic correlation to find the best topology (*e*).

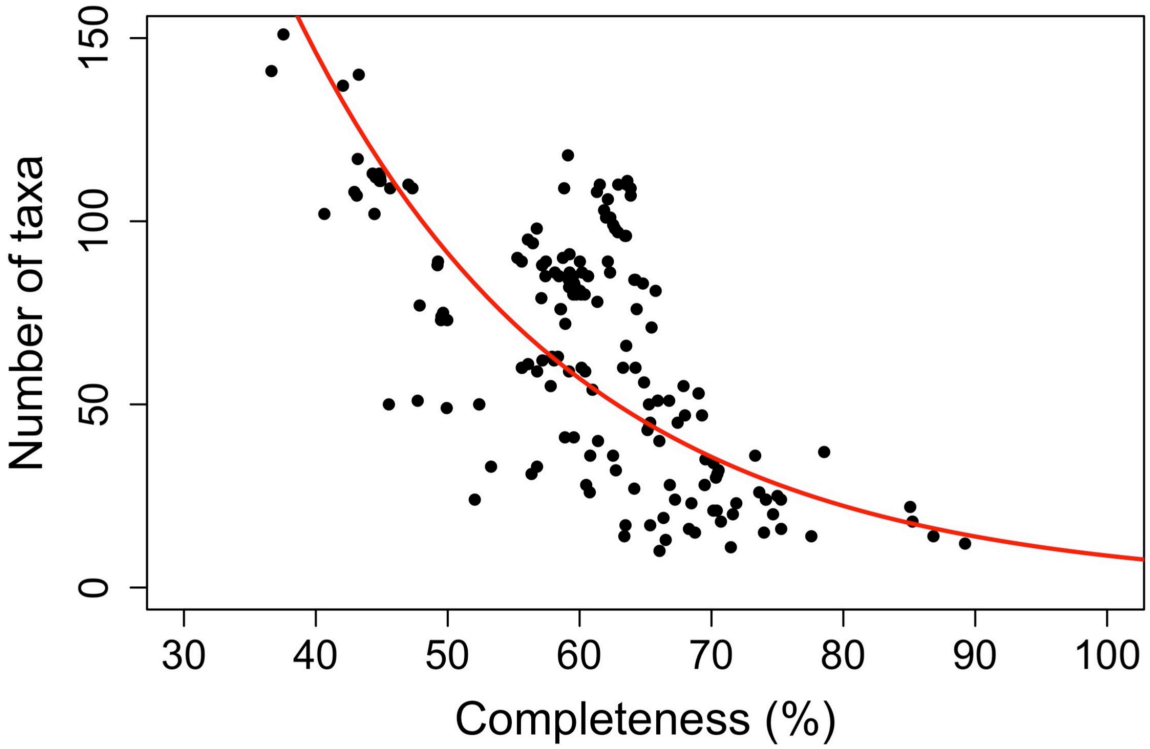

Fig. S2: Scatterplot indicating the diminishing completeness of large matrices. More inclusive matrices must enumerate a greater number of morphological characters in order for taxa to be distinguished. Inevitably large matrices include characters that are not applicable to the entire sample. As the number of taxa increases, so does the number of inapplicable characters, and therefore the relative completeness of the matrix declines.

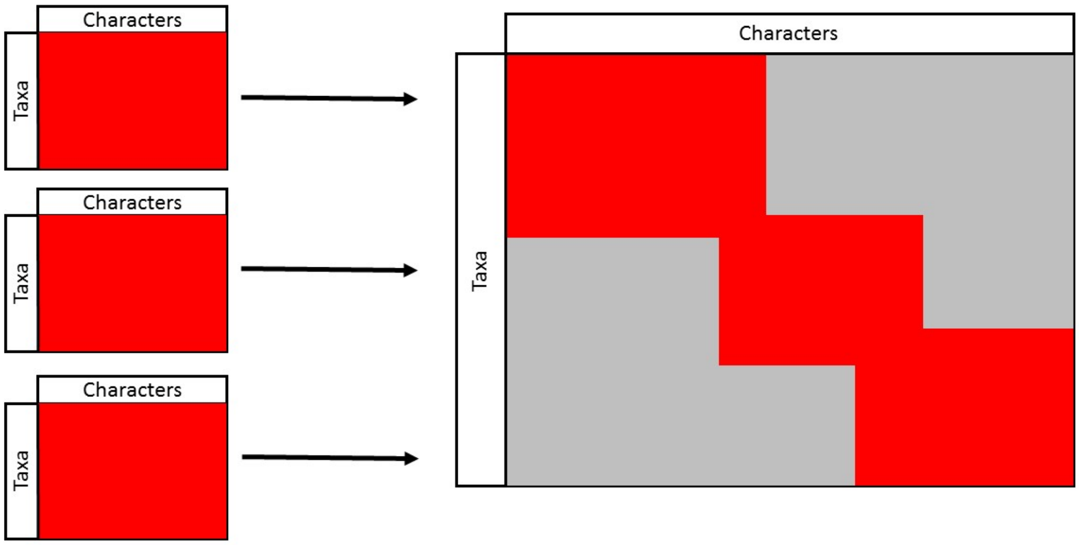

Fig. S3: Stylised representation showing the accumulation of missing data in supermatrices. Encoded characters are indicated in red, missing data in grey. Characters will not be applicable to all taxa. Therefore, as the number of source matrices increases the fraction of missing data also increases.

Fig. S4 (next page): Phylogenetic tree showing the outcome of the supertree analysis, pruned to fit the available body size trait data. This is a formal supertree estimated from a sample of source trees using the matrix representation with parsimony (MRP) method. The source trees were sampled from peer-reviewed publications since 2010, and re-analysed using Bayesian inference. The best supertree output by the MRP method was determined using stratigraphic correlation.

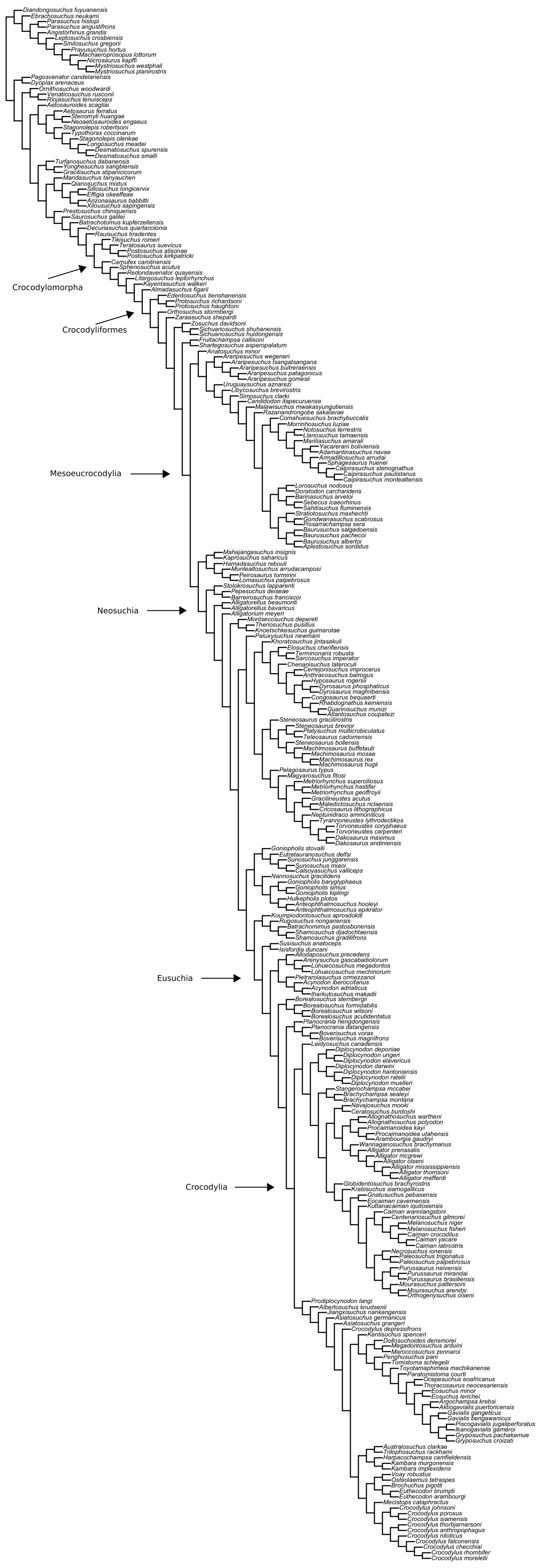
Crocodylomorpha
Crocodyliformes
Mesoeucrocodylia
Neosuchia
Eusuchia
Crocodylia
