# Supplementary material for: Environmental drivers of body size evolution in crocodile-line archosaurs
Source: Commun Biol. 2021 Jan 7;4:38. doi: 10.1038/s42003-020-01561-5 (PMC7790829; doi:10.1038/s42003-020-01561-5)
Supplement: Supplementary file 3 — Reporting Summary [file 42003_2020_1561_MOESM3_ESM.pdf]

nature research

Corresponding author(s): Maximilian Thomas Stockdale

Last updated by author(s): Nov 23, 2020

Reporting Summary

Nature Research wishes to improve the reproducibility of the work that we publish. This form provides structure for consistency and transparency in reporting. For further information on Nature Research policies, see Authors & Referees and the Editorial Policy Checklist .

Statistics

For all statistical analyses, confirm that the following items are present in the figure legend, table legend, main text, or Methods section.

n/a	Confirmed	
☐	☒	The exact sample size (*n*) for each experimental group/condition, given as a discrete number and unit of measurement
☐	☒	A statement on whether measurements were taken from distinct samples or whether the same sample was measured repeatedly
☐	☒	The statistical test(s) used AND whether they are one- or two-sided<br>*Only common tests should be described solely by name; describe more complex techniques in the Methods section.*
☒	☐	A description of all covariates tested
☐	☒	A description of any assumptions or corrections, such as tests of normality and adjustment for multiple comparisons
☐	☒	A full description of the statistical parameters including central tendency (e.g. means) or other basic estimates (e.g. regression coefficient) AND variation (e.g. standard deviation) or associated estimates of uncertainty (e.g. confidence intervals)
☐	☒	For null hypothesis testing, the test statistic (e.g. *F*, *t*, *r*) with confidence intervals, effect sizes, degrees of freedom and *P* value noted<br>*Give P values as exact values whenever suitable.*
☐	☒	For Bayesian analysis, information on the choice of priors and Markov chain Monte Carlo settings
☒	☐	For hierarchical and complex designs, identification of the appropriate level for tests and full reporting of outcomes
☒	☐	Estimates of effect sizes (e.g. Cohen's *d*, Pearson's *r*), indicating how they were calculated

Our web collection on statistics for biologists contains articles on many of the points above.

Software and code

Policy information about availability of computer code

Data collection	Data was collected by hand from published literature
Data analysis	Analyses were performed using self-contained software (BayesTraits, PaSt).

For manuscripts utilizing custom algorithms or software that are central to the research but not yet described in published literature, software must be made available to editors/reviewers. We strongly encourage code deposition in a community repository (e.g. GitHub). See the Nature Research guidelines for submitting code & software for further information.

Data

Policy information about availability of data

All manuscripts must include a data availability statement. This statement should provide the following information, where applicable:

- Accession codes, unique identifiers, or web links for publicly available datasets
- A list of figures that have associated raw data
- A description of any restrictions on data availability

All data analyzed in this study are taken from published sources, including all images of skeletal elements and NEXUS files containing phylogenetic matrices. These published sources are referenced in the Supplementary Information, document 1 and 2. All data generated in this study are included in the Supplementary Information, document 2. First- and last-appearance data was accessed from the Paleobiology Database (paleodb.org), and all these data are reproduced in the Supplementary Information, document 2.

Field-specific reporting

Please select the one below that is the best fit for your research. If you are not sure, read the appropriate sections before making your selection.

✘

Ecological, evolutionary & environmental sciences study design

All studies must disclose on these points even when the disclosure is negative.

Study description	Phylogenetic models of body size evolution in crocodile-line archosaurs using data from fossils
Research sample	Skull, mandibular, humeral and femoral characters collected from images of crocodile line archosaurs, dating from the Triassic period until the present day. A total of 280 taxa were collected, characterized by up to 21 traits depending on completeness.
Sampling strategy	The objective was to create as comprehensive a dataset as possible, so every available specimen was sampled.
Data collection	Data was collected from the literature, by measuring images in peer-reviewed publications
Timing and spatial scale	Data was collected from 2018-2019. The aim was to be as comprehensive as possible, collecting data from as many fossil taxa as possible, from every locality and geological stratum available.
Data exclusions	No data was excluded from the analysis.
Reproducibility	All methods are described comprehensively in the manuscript. All software is freely available. All data is included in the supplementary information.
Randomization	This analysis was of the total-group pseudosuchia, arranging into groups was not necessary.
Blinding	Blinding was not necessary for this study because the data points pertain to living interpretations of fossil specimens that cannot be observed directly.

Did the study involve field work? ☐ Yes ☒ No

Reporting for specific materials, systems and methods

We require information from authors about some types of materials, experimental systems and methods used in many studies. Here, indicate whether each material, system or method listed is relevant to your study. If you are not sure if a list item applies to your research, read the appropriate section before selecting a response.

Materials & experimental systems

n/a	Involved in the study	
☒	☐	Antibodies
☒	☐	Eukaryotic cell lines
☐	☒	Palaeontology
☒	☐	Animals and other organisms
☒	☐	Human research participants
☒	☐	Clinical data

Methods

n/a	Involved in the study	
☒	☐	ChIP-seq
☒	☐	Flow cytometry
☒	☐	MRI-based neuroimaging

Palaeontology

Specimen provenance	Fossils were sampled from images in the literature. All the images sampled are cited in the supporting information.
Specimen deposition	Specimens are worldwide in spatial range.
Dating methods	Dating methods are discussed in the source publications cited in the supplementary information.

☒ Tick this box to confirm that the raw and calibrated dates are available in the paper or in Supplementary Information.
